# A systematic review of second-hand smoking mass media campaigns (2002–2022)

**DOI:** 10.1186/s12889-024-18222-5

**Published:** 2024-03-04

**Authors:** Carmen C.W. Lim, Brienna Rutherford, Coral Gartner, Caitlin McClure-Thomas, Shaun Foo, Fang-Yi Su, Roman Scheurer, Susy Sebayang, Gary Chan, Daniel Stjepanović, Fitri Fausiah, Ghea Farassania, Janni Leung

**Affiliations:** 1 National Centre for Youth Substance Use Research, The University of Queensland, 31 Upland Road, 4072 St Lucia, QLD Australia; 2 School of Psychology, The University of Queensland, Brisbane, Australia; 3 NHMRC Centre of Research Excellence on Achieving the Tobacco Endgame, School of Public Health, The University of Queensland, Brisbane, Australia; 4 Queensland Centre for Mental Health Research, The Park Centre for Mental Health, Wacol, Australia; 5 School of Public Health, Universitas Airlangga, Surabaya, Indonesia; 6 School of Psychology, Universitas Indonesia, Depok, Indonesia

**Keywords:** Second-hand smoking, Passive smoking, Mass-media campaign, Interventions

## Abstract

**Background:**

Second-hand smoking (SHS) increases the risk of chronic disease in adults and poses a serious health threat to children. Mass media campaigns are instrumental in raising awareness and reducing SHS exposure. There is a need to identify recent SHS mass media campaigns and assess their sustainability in terms of knowledge, attitudes, and behavioural changes. This systematic review summarises the characteristics and outcomes of mass media campaigns on SHS prevention.

**Methods:**

PubMed, Embase, Web of Science, and grey literature were searched in November 2022 for SHS campaigns implemented between 2016 and 2022. The eligibility criteria included campaigns on the dangers or effects of SHS with any target group, dissemination medium, study design, or language. The database search identified 1,413 peer-reviewed titles, of which 82 full-texts were screened, with 14 meeting the eligibility criteria. The grey literature search identified 9,807 sources, of which 61 were included. We extracted data on the campaign characteristics, metrics, and smoking-related outcomes. The JBI critical appraisal tool was used to assess the risk of bias of the included studies.

**Results:**

We found 73 SHS campaigns conducted between 2002 and 2022, across 50 countries. The campaigns reached 378 million people. The reported recall rates range from 8 to 76%. Of the 11 studies that reported smoking-related outcomes, 10 reported increased knowledge in understanding SHS risks (73-85%), five reported an increased prevalence of smoke-free homes, and two reported an increase in number of participants persuading others to quit smoking. Two studies reported a decrease in overall smoking, whereas three studies observed a reduction in smoking in the presence of children.

**Conclusion:**

The available data provide some support for the effectiveness of SHS campaigns in reducing smoking behaviours in homes and around children. However, the certainty of evidence was low due to the lack of a control group and the substantial heterogeneity in the outcomes assessed. Future campaigns need comprehensive evaluation and reporting to reduce publication bias.

**Supplementary Information:**

The online version contains supplementary material available at 10.1186/s12889-024-18222-5.

## Introduction

Second hand smoking (SHS) increases the risk of chronic physical conditions in adults, while also posing severe health risks (e.g., sudden infant death syndrome) to children [1–5]. The 2019 Global Burden of Disease Study estimated that exposure to SHS contributed to 1.3 million premature deaths among non-smoking individuals globally, with 50,000 of these deaths occurring in children below 14 years [6]. SHS exposure is prevalent in countries with a high prevalence of adult smoking [7]. Data from the Global Youth Tobacco Survey, involving 711,366 participants from 142 countries found that 33.1% of adolescents had been exposed to SHS at home, and 57.6% in public places [8]. SHS exposure was also reported in other settings such as vehicles, school, and workplaces [8]. 

The WHO’s MPOWER package, comprising six measures, [9] incorporates two crucial elements that align with mass media campaigns: ‘*Protect people from tobacco smoke*’ and ‘*Warn about the dangers of tobacco*’. These components, when integrated with broader tobacco control strategies like enforcing smoke-free policies, significantly enhance the effectiveness of mass media campaigns in reducing population-level tobacco use [10, 11]. Mass media campaigns targeting SHS are instrumental in raising awareness about its harmful effects, reducing exposure, altering attitudes and beliefs among people who smoke, and promoting smoking cessation [12]. However, 88 countries (56% of the world’s population) have not implemented any comprehensive national anti-tobacco campaigns (including anti-SHS campaigns) [11]. 

Earlier seminal work [13] that evaluated campaigns from 9 countries found that messages that highlighted the health dangers of SHS can serve as catalysts for behavioural change. These messages not only educate those who are smoking about the dangers they pose to others, but also tap into their concern for those around them. Another seminal work by Tobacco-Free Kids, an American non-profit organisation, has synthesised data on 30 national- or state-level SHS campaigns from 17 countries implemented between 2000 and 2009 [14]. They identified several key success factors that could influence audiences’ knowledge and attitudes, including utilising personal stories and content that communicate the dangers of SHS, including those that demonstrate the impact of SHS on children, and avoiding content that demean people who smoke [14]. A 2017 Cochrane review that evaluated the effectiveness of tobacco control mass media campaigns published up to 2016 (including campaigns that focused on SHS) found such campaigns to be effective in increasing the knowledge about the harms of SHS [15]. There is a need to assess the sustainability of the campaign effects on knowledge, attitudinal shifts, the establishment of smoke-free homes, and behavioural changes over a period of time after the campaign has concluded.

The rise of digital media platforms such as YouTube and Instagram has changed the way people access and interact with the media. In addition to traditional media, some countries [16, 17] are now leveraging digital platforms to increase reach and target their campaigns to those who are most at risk, resulting in a broad array of measures and metrics utilised in each campaign, making comparisons between campaigns difficult. To guide the reporting of campaign evaluations, Chan et al. developed a conceptual framework for evaluating antitobacco campaigns. This framework is structured around different levels of evaluation, including process (how the campaign is delivered), impact (awareness, engagement, and initial behavioural change), and outcome (focusing on the desired behavioural changes).

The aim of this systematic review is two-fold: (1) to identify and describe recent mass media campaigns on SHS globally, including campaigns deployed over social media and traditional media channels launched after 2016; and (2) to summarise the post-campaign related metrics and evaluations, such as reductions in SHS exposure, using the conceptual framework laid out by Chan and colleagues [18].

## Methods

This review followed the Preferred Reporting Items for Systematic Review and Meta-analyses (PRISMA) guidelines (Table S1) and was registered on PROSPERO (CRD42022322843).

### Eligibility criteria

The inclusion criteria were: (1) campaigns that focused on SHS or its harmful effects; (2) city, regional, or national campaigns targeting any population; (3) reports detailing at least one aspect of the campaign (e.g., aims, key messages, post-campaign outcomes); (4) mass media campaigns deployed through social media or traditional media; (5) any study designs; or (6) any language. Campaigns that did not report on post-campaign outcomes were still eligible and used to provide information on the characteristics of recent SHS campaigns. Exclusion criteria included: (1) general anti-smoking campaign not specific to SHS; (2) interventions to reduce SHS (e.g., counselling sessions or community centre classes); (3) pre-campaign studies that informed the development of the SHS campaign but did not evaluate or describe the final campaign.

### Search strategy

Our search strategy involved two steps: (1) database search of PubMed, EMBASE, and Web of Science; (2) grey literature search. Three databases (PubMed, EMBASE, and Web of Science) were searched for peer-reviewed literature or conference abstracts published between 2016 and 2022 in April 2022 using search terms related to SHS, media, and behavioural outcomes. This timeframe was chosen because a related review [15] had comprehensively evaluated the literature until 2016. It is worth noting that while our search focused on literature published between 2016 and 2022, some of these studies evaluated campaigns that were initiated prior to 2016. A specialist research librarian was consulted to finalise the search strategy (Table S2). In addition, the reference lists of each included study and email alerts from relevant journals until December 2022 were manually searched for potential studies by a single author (CL). The search results were exported into EndNote [19] to remove duplicates, and the final list was uploaded into Covidence [20] for screening.

A grey literature search was conducted between April and December 2022 to identify SHS campaigns that were not found in peer-reviewed literature. This involved searches on tobacco control organisation websites, the YouTube platform, the Factiva database for news articles, [21] Google Advanced Search, and consulting with subject experts.

### Screening

The title and abstracts of peer-reviewed literature were independently screened by at least two reviewers (CL, JL, CM, and DS). Full-text articles were independently assessed for eligibility by at least two reviewers (FY, SF, CL, and JL). Any disagreements were resolved by a third reviewer, who was not involved in the initial screening. The corresponding authors of the conference abstracts were contacted, where in-depth details about the study were not publicly available. Eligible studies were assessed by at least two reviewers (CL, FY, SF) for any potential risk of bias using the JBI critical appraisal tool [22]. 

To assess the eligibility of the grey literature, all documents were independently screened by at least two reviewers (CL, FY, SF, and RS) according to the inclusion and exclusion criteria. For Google Searches, only titles listed on the first 3 pages (25 results list) were assessed for eligibility. Other grey literature materials (e.g., executive summaries, abstracts, news articles, YouTube short videos) were reviewed in full.

### Data extraction

The following were extracted from each article: 1) author, year, campaign or policy name, year of implementation, scale of the campaign, country (with site details), target audience, materials used in the campaign, media channels, responsible agency/organisation, and post-campaign evaluation (if any).

### Synthesis of results

**Fig. 1:**
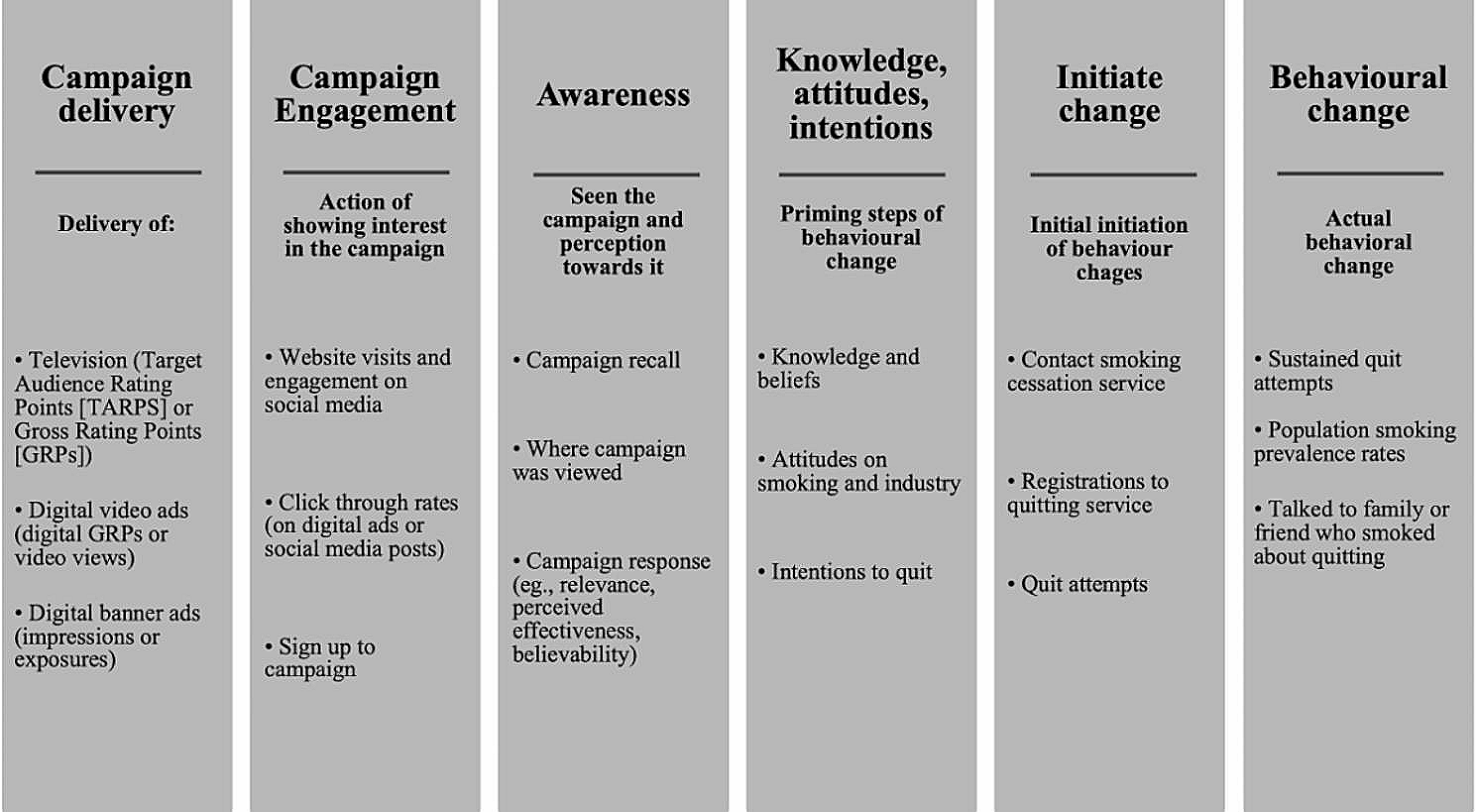
Campaign evaluation metrics, adapted from Chan et al. [18]

We used Chan et al’s [18] conceptual framework to evaluate each campaign. This framework comprises three primary components: (i) process evaluation (*campaign delivery*); (ii) impact evaluation (*campaign engagemen*t; *campaign awareness*; *knowledge, attitudes, and intentions*; *initiate change*), and (iii) outcome evaluation (focusing on actual post-campaign *behavioural changes* such as sustained quit attempts) (Fig. 1). Within the impact evaluation component, four sub-forms were utilised: ‘*awarenes*s *of campaign*’ assessed participant’s perception towards and ability to recall the campaign; ‘*campaign engagement*’ measured how individuals interacted in response to the campaign; and ‘*knowledge, attitudes, and intention’* evaluated changes in knowledge, attitudes and beliefs; and ‘*initiate change*’ assessed the extent to which individuals made efforts to quit smoking.

## Results

### Study selection

**Fig. 2:**
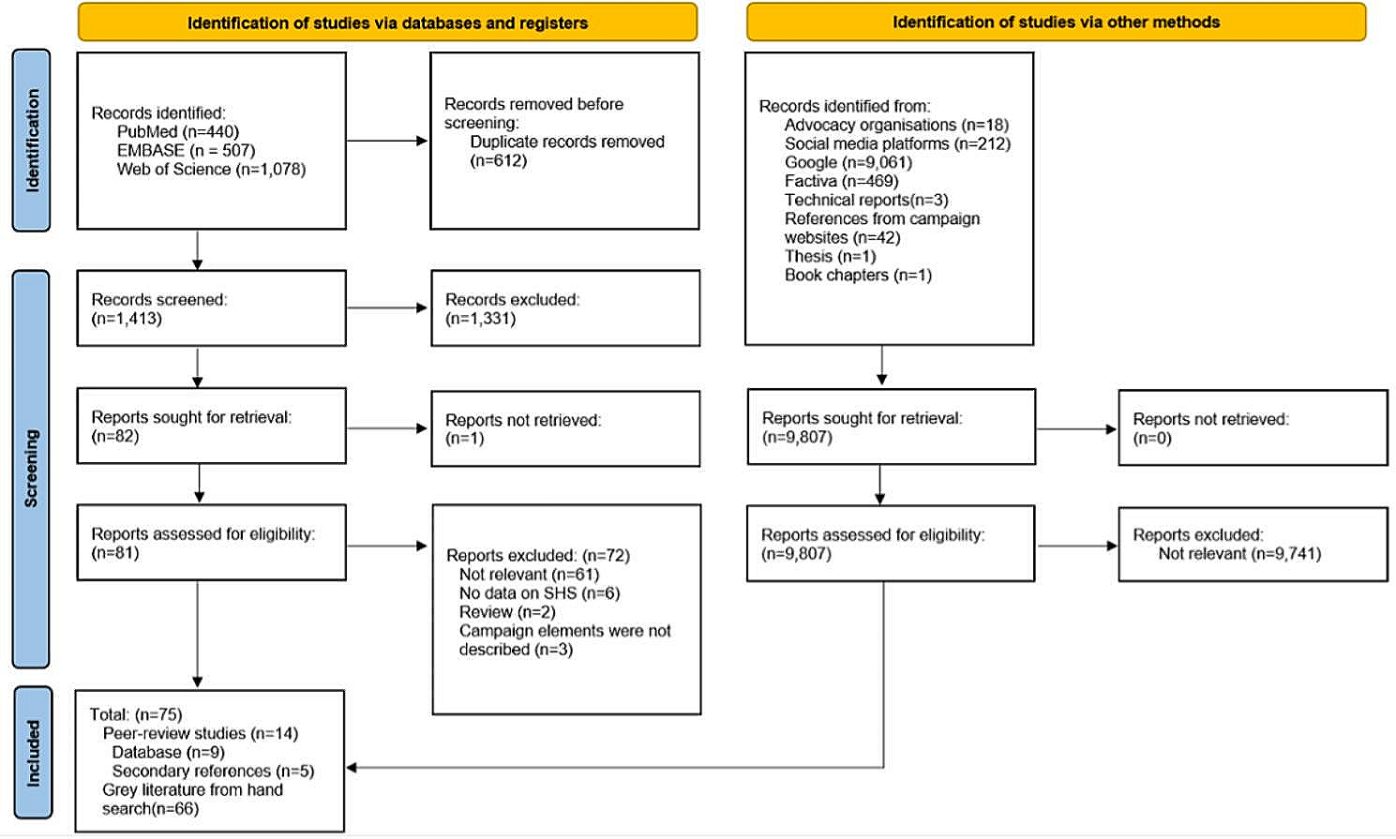
PRISMA diagram

This study identified 1,413 peer-reviewed articles and conference abstracts. After screening their titles and abstracts, we screened the full text of 81, ultimately including 9 articles that met the inclusion criteria and 5 eligible publications from screening the reference lists (Fig. 2). The 14 included papers, [23–36] reported 13 mass-media campaigns implemented between 2002 and 2019 in eight countries (Vietnam, China, U.S., England, Scotland, Indonesia, Bosnia and Herzegovina, and Ukraine). These studies [23–36] evaluated mass media campaigns using pre-and post-campaign surveys or semi-structured interviews, [23–29, 34] ecological studies (e.g., hospital admissions, Quitline call volume data), [33, 35] not clearly described, [30, 31] or social media metrics data [32, 36]. The grey literature search located 61 mass media campaigns [12, 17, 37–95] from 44 countries implemented between 2016 and 2022. Both peer-reviewed and grey literature searches identified 75 mass media campaigns (73 unique campaigns from 50 countries), since two campaigns were covered in grey literature [43, 59] and peer-reviewed literature. Among peer-reviewed publications, the risk of bias assessment for cross-sectional studies ranged from 5 to 8 (out of 11), indicating a medium to low risk of bias, while cohort [23] and qualitative studies [27, 36] had a medium risk of bias (Table S3).

### Campaign characteristics

**Fig. 3:**
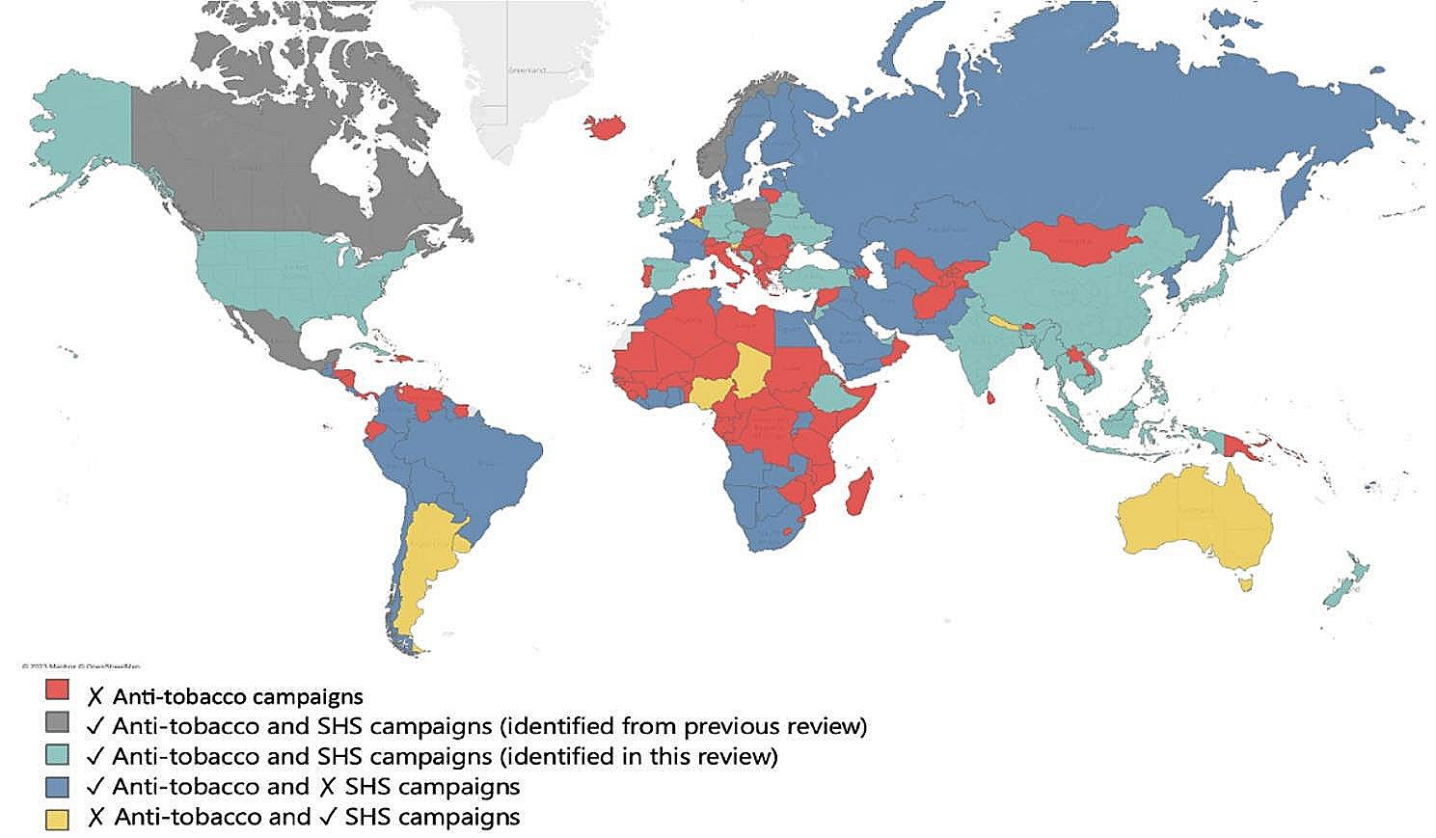
Anti-tobacco campaigns (2018–2020)^a^ and second-hand smoking campaign coverage (2002–2022)

All campaigns aimed to increase awareness of SHS to protect children from the harm of SHS and/or comply with smoke-free laws. The campaigns were implemented across all WHO regions with less coverage from South America, Middle Eastern, and African regions (Fig. 3). Forty-one campaigns were implemented on a national scale, 31 campaigns on a subnational (state/city/district/county) scale, and one on a global scale.

### Campaign delivery

Of the 73 included campaigns, 47 disseminated content online or via social media platforms, such as Facebook and YouTube. Traditional print media (*n* = 19) and television (*n* = 16) were common distribution channels. An equal number of campaigns (*n* = 12) used radio and billboard/poster campaign delivery, and community events were used to spread awareness of the campaign objectives. The target population of these campaigns is people who smoke, women or the general population. Most campaigns use emotional narratives, such as the testimonials of victims or victims’ families, to increase awareness (see Table 1). Additional details can be found in Tables S4 and S5.

**Table 1:** Sample characteristics of second-hand smoking campaigns

Type of evaluation	Number of campaigns	Scale	Country (Reference)	Year	Metrics or findings	Conclusion
1) Campaign delivery	74	C, A, S	Vietnam [28, 40, 55, 82], China [29, 56, 94], UK [24, 25, 27, 31, 43, 72, 80], US [23, 26, 33, 38, 48], Indonesia [30, 36], Bosnia and Herzegovina [32], Ukraine [34, 59], United Arab Emirates [86], Cuba [90], Jordan [89], India [50, 92], Japan [69, 87], Qatar [91], Luxembourg [88], Nepal [93], New Zealand [51, 62], Belarus [74], Hong Kong [77], Malaysia [17, 63, 79], Argentina [47], Turkey [82], Bangladesh [49, 83], Ireland [68, 70, 71, 75, 78, 85], Ethiopia [81], Australia [61, 73], Myanmar [64], Czechia [65], Thailand [12], Nigeria [60], Bahamas [58], Trinidad and Tobago [67], Slovenia [57], Bosnia and Herzegovina [53], Belgium [95], Spain [52], Austria [54], Armenia [45], Cambodia [42], Philippines [46], US Virgin Islands [44], Malta [76],Germany [37], Tonga [39], Chad [41], Global [66]	2002–2022	All but one campaign [35] described campaign delivery. Most campaigns (64%) disseminated their content online	---
2) Campaign engagement	25	C	Bosnia and Herzegovina [32, 53], United Arab Emirates [86], New Zealand [51, 62], Hong Kong [77], UK [72, 80], Turkey [82], Bangladesh [49, 83], Malaysia [17], Japan [69], Australia [61, 73], India [50], China [56, 94], Cambodia [42], Philippines [46], US [48], Tonga [39], Indonesia [36], Vietnam [28], Chad [41]	2014–2022	19 campaigns reported social media engagement metrics, with 3 reporting views in the millions. Two campaigns reported in-person attendance as the engagement metric of choice, ranging between 350 and 10,000 attendees. Three further campaigns reported population reaches of 3.481 million (Bosnia and Herzegovina), 329 million (China), and 378 million (US).	Yes, effective
3) Awareness of campaign	6	C, A, S	Vietnam [28], UK [27, 43], US [23, 26], Indonesia [36]	2002–2018	All campaigns evaluated recall or recognition without prompt, with recall ranging from 8.0-76.4%. Limited evidence showed factual (vs. personal stories) and gain-framed (vs. loss-framed) campaigns resulted in better recall.	Mixed
4) Knowledge, attitudes, and intentions	10	C, A, S	Vietnam [28], China [29], UK [24, 25, 27, 43], Indonesia [30], US [23], Ukraine [34], Hong Kong [77]	2002–2021	Ten campaigns found increased awareness of SHS dangers and increased intention to quit, however high intentions to quit did not necessarily correlate with actual behavioural change. Notably, one campaign identified that respondents sought to quit, it was not attainable in their current circumstances.	Mostly effective
5) Initiate change	8	C, A, S	Vietnam [28], China [29], UK [27, 43], US [23, 33], Ukraine [34], Malaysia [79]	2002–2021	Two campaigns found an increase in quit attempts among respondents. However, a third found exposure to SHS campaigns did not predict quit attempts. An additional five campaigns found increased support of public smoking bans, commitments to smoke-free homes, increased pressure to quit, transition to alternate consumption and increased Quitline calls.	Mixed
6) Behavioural change	7	C, A, S	Vietnam [28], UK [24, 25, 35, 43], US [23], Ukraine [34]	2002–2019	Three campaigns assessed whether smoke-free promises were maintained post-campaign. Each campaign reported increases in smoke-free households and decreases in children’s exposure to SHS. Two campaigns found 77% of Vietnamese and 30.2% of Ukrainian respondents indicated they had attempted to persuade others to quit or commit to smoke-free homes. Two remaining campaigns found no significant association between campaign engagement and smoking abstinence.	Effective for smoke-free homes No effect for smoking abstinence

*Abbreviations* G: Global; C: Country-level; S: State-level; A: Area/District/County-level

*Source*^*a*^: *The World Health Organisation report on the global tobacco epidemic 2021: addressing new and emerging products*

### Campaign engagement

Engagement (how individuals interacted in response to the campaign) was assessed in various ways depending on the dissemination channel. Twenty-five campaign evaluations included at least one measure of engagement, with social media metadata (e.g., ‘views’, ‘likes’, ‘followers’, and ‘retweets’) being the most reported metric.

Three evaluations reported views in the millions. Two campaigns launched in 2016 in Vietnam and the U.S. received over 4 million views on Facebook [28] and YouTube [48], respectively. A similar campaign launched on Weibo reported more than 5.8 million views on their SHS advertisements [56]. Another 16 social media campaign evaluations [17, 36, 39, 41, 49–51, 61, 62, 69, 72, 73, 80, 82, 83, 94] that reported views had an average of 39,185 views on YouTube, with a range of 147 [17] to 530,000 [62] (Table S6).

Of the three social media campaign evaluations that reported the number of followers as their engagement metric, an ongoing Facebook campaign based in Bangladesh had the highest reported follower count at 766,000 [49]. The remaining two Facebook campaigns based in the Philippines and Hong Kong had approximately 6,000 [46] and 7,500 [77] followers, respectively as of writing.

Two campaign evaluations reported attendance numbers as their chosen engagement metric. A Cambodian campaign involving public seminar talks recorded 350 attendees, [42] while a 2018 creative exhibit held in Bosnia and Herzegovina attracted over 10,000 attendees [53]. An additional campaign evaluation that reported program registration as the engagement metric, recorded 107 enrolments in the Malaysian MyHOUSE smoke-free environment campaign [79]. 

The final three campaign evaluations used population reach as the engagement metric. The earliest campaign of the three, was a 2014 Chinese television campaign which reached 24% of the total population (329 million people) [29]. A 2016 US social media campaign reached 378 million people, exceeding the population of the US in 2016 [96] suggesting the possibility of repeated exposure to the same individuals. Similarly, a 2016 social media campaign [32] based in Bosnia and Herzegovina reported reaching more than 3 million people, which almost equalled the 2016 population total of 3.481 million [97]. 

In summary, SHS campaigns have been increasingly delivered through social media, with engagement metrics supporting a noticeable reach.

### Awareness of campaign

Six campaigns measured recall or recognition of campaign messages without prompting in post-campaign-exposure surveys [23, 26–28, 36, 43]. Campaign recall ranged from 8.0% [28] to 76.4% [27], with an average of 44.4% of respondents being able to recall campaign messaging without prompting. The lowest recall score was for a Vietnamese campaign which used a 30-second testimonial of a 41-year-old lung cancer victim to spread awareness on social media, radio, and television [28]. The highest recall score was reported for a Scottish campaign that depicted the dangers of SHS with facts (e.g. ‘*second-hand smoke damage can continue up to 5 hours after a cigarette is extinguished*’) [27]. It is interesting to note that despite respondents reporting testimonials to be more emotionally impactful (as indicated above), these campaigns were less recalled than their more factual counterparts.

Of these campaigns, two campaigns [26, 36] assessed viewer perceptions of smoke-free campaign messaging-loss-framed (i.e., demonstrating the risks of SHS) versus gain-framed (i.e., demonstrating the benefits of smoke-free environments) advertisements. A 2010 U.S. based campaign found that loss-framed advertisements had higher emotional impact than gained framed advertisements (68% vs. 58%, *p* < 0.001) but gain-framed advertisements were more likely to be recalled than loss-framed advertisements (recalled by 29% and 20%, respectively) [26]. Additionally, an evaluation of an Indonesian anti-smoking YouTube campaign found that loss-framed advertisements that featured stories of victims or their families to increase awareness of SHS dangers were more acceptable than advertisements that displayed graphic images of tobacco-related disease [36]. 

In summary, limited evidence has shown that factual (vs. personal stories) and gain-framed (vs. loss-framed) campaigns result in better recall, but the effectiveness of these campaigns may depend on the geographical or cultural context of the target audience.

### Knowledge, attitudes, and intentions towards SHS

The evaluation data included 10 campaign evaluations measuring knowledge or belief-related outcomes, such as increased awareness of the associated harm and benefits of smoke-free homes. These outcomes are typically measured using post-engagement interviews or as part of an evaluation.

Several post-campaign evaluations have found an increased awareness of the dangers of SHS. A 2002 U.S. campaign found that recall of SHS advertisements was associated with higher awareness of the harmful effects of breathing in SHS (OR:2.1, 95% CI:1.1-4.0) [23]. Similarly, a 2009 English campaign found 77.7% of respondents had increased awareness or concern about the risks of SHS [25]. A review of the effectiveness of a 2014 Chinese television and social media campaign found increased awareness of the harmful effects of SHS and increased knowledge that smoking causes diseases other than lung cancer (73% and 85%, respectively) regardless of smoking status [29]. These findings were mirrored in a 2017–2018 English campaign evaluation, which found that 75% of respondents were more concerned about smoking after viewing the campaign message [43]. 

Five campaign evaluations assessed the intention to quit smoking, using a post-engagement survey. Evaluations of two 2014 campaigns, one in Scotland [27], and the other in China, [29] found that those who engaged in the campaign had increased intentions to quit. The intention to quit smoking and remove SHS from homes were also key outcomes for two U.K. community-based smoke-free-home campaigns, [24, 43] and an Indonesian campaign implemented between 2015 and 2018 [30]. A similar promise to action was noted in a 2021 Hong Kong social media campaign, where over 500 citizens pledged to encourage those around them to refrain from smoking [77]. However, high intentions to quit did not necessarily correlate with actual behavioural change (e.g., sustained quit attempts). The majority of viewers of a 2014 Scottish campaign thought smoke-free homes were desirable and wanted to quit smoking but did not believe it was attainable in their current circumstances [27]. 

In summary, the evaluated campaigns were effective in increasing knowledge of the harms of SHS, intentions to quit, and smokefree homes.

### Initiate change

Eight campaign evaluations revealed that individuals made efforts to quit smoking, increase support for smoking bans, call volumes to Quitline, or transitioned to other methods of consuming nicotine after exposure to the campaign.

Three of these campaigns assessed quit attempts [23, 28, 43]. A 2016 social media campaign evaluation in Vietnam found that 74% of women who don’t smoke and 75% of men who smoke had attempted to make their households smoke-free and a further 67% of men who smoke reported quit attempts [28]. An English campaign focusing on reducing children’s SHS exposure the following year also found that 38% of the target audience attempted to reduce smoking behaviours after viewing the campaign [43]. However, a 1-year follow-up study found exposure to SHS advertisements did not predict quit attempts (OR:1.3, 95% CI:0.8–2.1) [23].

Other evaluation studies found that support for bans in public places increased after the campaign, particularly among non-smoking individuals, [29] while those who smoke reported feeling increased pressure not to smoke at home and at public places [34]. Some individuals switched to other methods of nicotine consumption (e.g., e-cigarettes) [27]. Additionally, a U.S. study revealed that increased exposure to Spanish-language ads that highlighted the health effects of SHS was associated with an increased call volume to a Spanish language Quitline [33]. Another ongoing campaign, MyHouse, has resulted in 107 Malaysian households committed to making their homes smoke-free [79]. 

In summary, these campaigns demonstrated increased efforts to quit smoking. Support for public smoking bans and an increase in smoking cessation aids and service use have also been reported in campaign evaluations.

### Behavioural change

Seven campaigns reported evaluations of desired or actual changes among campaign audiences. Five campaigns assessed actual behavioural change (i.e., sustained quit attempts and maintained a smoke-free home [23–25, 28, 35, 43]. An evaluation of the English campaign, *Smoke-Free Homes*, found that 90% of involved households maintained their smoke-free promise and that the proportion of households that reported being smoke-free increased from 35% at baseline to 68% in the six-months following the campaign [24]. Another English *Smoke-Free Home* campaign evaluation found that 78% of households became smoke-free after engaging with the campaign [25]. The third campaign, the Scottish *Take it Right Outside* program, found that children’s SHS exposure decreased from 12% two years prior to the campaign (2012), to 6% in the year following the campaign (2015) [35]. This evaluation also noted a decrease relative to the underlying slope in hospital admissions for asthma among younger children (-0.48%, (-0.85 to -0.12)), *p* = 0.0096), suggesting improved health outcomes from decreased SHS exposure [35]. Although campaign exposure were linked to quit attempts, [28, 43] a 2002 US based campaign featuring testimonials reported no significant association between viewing the campaign materials and smoking abstinence at 12-month follow-up [23]. 

A further two campaigns assessed desired behavioural change in the context of persuading others to quit or commit to smoke-free areas [28, 34]. Vietnam’s *Women Create Smoke-Free Homes* campaign, which launched in 2016, reported 77% of respondents indicated they had attempted to persuade others to quit smoking after engaging with the social media campaign [28]. Similarly, a 2019 Ukrainian campaign reported 30.2% of respondents had protested others smoking in designated smoke-free areas, including talking to the person smoking, posting signs, and reporting the incident to authorities [34]. 

In summary, SHS campaigns increased smoke-free households, reduced children’s SHS exposure, and increased speaking out against SHS exposure, but not sustained quit attempts.

## Discussion

This systematic review provides a comprehensive and centralised resource of information on SHS prevention mass media campaigns worldwide, making it a valuable tool for researchers, policymakers, and public health professionals to reduce the burden of disease attributable to SHS. We found 73 SHS campaigns conducted between 2002 and 2022 across 50 countries, most of which were implemented in countries with high smoking prevalence. Very few campaigns have been conducted in from South America, Africa, and the Middle East. A key strength of the current review is the comprehensive literature and an extensive search of grey literature (> 9000 documents). We identified only 14 mass media campaigns in the peer-reviewed literature, but an additional 59 campaigns were identified in the grey literature.

We found most campaigns, particularly those from low-middle income countries, deployed media and advertisements that featured emotional narratives with hard hitting tones (i.e., loss framed messages), [28, 36, 40, 41, 49, 62, 84] a typical strategy used in tobacco control to promote non-smoking behaviours [98]. Loss-framed advertisements in SHS campaigns typically depicted the dangers of SHS through the personal stories of people with cancer and involved a call to action to protect loved ones by not smoking. Loss-framed advertisements were also found to be more effective at prompting emotion than gain-framed advertisements [26]. Other campaigns [69, 80] deployed fewer graphic advertisements (e.g., animations and cartoons), which may resonate with younger age groups due to increased appeal. Different target audiences may respond differently to messages (children versus smoking adults), future campaigns need to consider the cultural and contextual factors in developing these messages [14]. 

Campaigns have increasingly integrated social media alongside traditional mass media mediums such as television and radio, leveraging the unique strengths of each to enhance reach, recall, engagement rate, and cost-effectiveness, which can lead to a more effective campaign [14]. For example, social media can be a cost-effective way to spread awareness on SHS while overcoming the limitations of traditional media by allowing for greater audience targeting (tailoring campaign to suit a specific demographic) and real-time engagement with the audience. Future campaigns should explore the utility of social media influencers in the dissemination of campaign messaging to increase their potential reach and acceptance of these messages. Existing literature has found that influencers engage in parasocial relationships with viewers, wherein these viewers tend to believe the influencer to be directly communicating with them [99]. Repeated interactions over an extended period lead to stronger para-social identification and an increased likelihood of viewers adopting the attitudes and behaviours of the influencer, [100] which could be beneficial in tobacco control campaigns.

Evaluation data were not available for most of the campaigns we identified, since most of the data came from the grey literature. Evaluation data can inform campaigns that have achieved their intended objectives and how different demographics are responding to the campaign, which could guide the future allocation of resources. In studies that provided evaluation data, the designs frequently lacked control or comparison groups, making it challenging to attribute the observed changes in behaviour or attitudes directly to the campaign. Additionally, there is a noticeable lack of longitudinal designs that would allow for the assessment of the sustained impact of campaigns over time. Despite this, the available evidence suggested that the campaigns were effective in increasing knowledge, increasing objecting to SHS exposure, and reducing SHS in the home, which could potentially shift societal norms towards anti-smoking and an increased community awareness of associated risks [101]. 

Although SHS campaigns show promise in reducing exposure among vulnerable populations, it is important for future campaigns to consider unintended consequences. One potential consequence can include stigmatisation of those who are already smoking [102, 103]. For example, the use of stigmatising language in the 1999 “*It’s Okay to Say You Mind*” campaign created a divisive “*us versus them*” mentality that could marginalise people who are smoking and reinforce stereotypes. Campaigns which are perceived as critical towards tobacco users are less effective in changing behaviours than those which employ positively geared language or slogans [14]. Those who feel that campaigns that fail to acknowledge prior quit attempts and associated difficulties or use confrontational narratives are more likely to reject campaign messaging due to marginalisation [14]. SHS campaigns need to be carefully designed and executed to have desired impact and mitigate unintended consequences [103]. Accordingly, it is important to follow the WHO recommendations of pre-testing campaigns with target audiences to refine the campaign objectives, and for campaigns to include an outcome evaluation to assess the impact [104].

### Limitations

Our search terms were limited to the English language, which may have inadvertently excluded studies published in other languages. Only limited process evaluation information was available on the campaigns; as such, we know little about the factors associated with their increased reach and success. For example, we do not know the cost (TARPS), level or frequency of exposure, tone and type of advertisements, media streams used, etc. for most campaigns. The absence of these data limits our ability to fully assess the reach and penetration of these campaigns and, consequently, their potential impact on awareness and behaviour change. In addition, the varying metrics collected in this review cannot be directly compared (e.g., TV metrics are not the same as digital metrics, or using view counts as a metric of awareness does not necessarily reflect the population exposed, as a proportion with a denominator would provide a more useful measure). While a campaign may have reported reach through media placement, it does not necessarily reflect the coverage of the entire population. Our findings may be subject to publication bias if the evaluations that showed negative results were not published. Many of the campaigns found in the grey literature did not report detailed post-campaign metrics (statistics). It is also challenging to attribute causality to the outcomes of the SHS campaigns because other factors such as new policies implemented at the same time (e.g., introduction of smoke-free laws) and pre-existing trends (e.g., smoking rates had already been declining in the population) may have contributed to the result. Reducing the prevalence of smoking and promoting of smoking cessation would translate to reduced exposure to SHS as well, but we do not have data to compare SHS exposure outcomes following general anti-smoking campaigns vs. SHS exposure-specific campaigns.

## Conclusion

Many mass media campaigns for SHS have been implemented in countries with a high tobacco burden. An increase in the number of campaigns delivered via social media is evident. Some evidence suggests that loss-framed advertisements are more acceptable but are associated with less recall, and the effectiveness of these campaigns may depend on the cultural context. The available evaluation data suggest that the campaigns were successful in raising awareness, encouraging speaking out against SHS, reducing smoking at home and in front of children, and increasing smoking cessation. However, the certainty of the evidence regarding the effectiveness of the campaign was low, primarily because of the absence of control groups, and the outcomes varied substantially across different studies. To improve future campaigns, it is crucial to undertake comprehensive pre- and post-campaign evaluations and report campaign implementation and evaluations to reduce publication bias.

### Electronic supplementary material

Below is the link to the electronic supplementary material.

Supplementary Material 1

## Data Availability

Data supporting the results can be found here: https://github.com/clim072/SHS.
