# Supplementary material for: A systematic review of second-hand smoking mass media campaigns (2002–2022)
Source: BMC Public Health. 2024 Mar 4;24:693. doi: 10.1186/s12889-024-18222-5 (PMC10913644; doi:10.1186/s12889-024-18222-5)
Supplement: Supplementary file 1 — Supplementary Material 1 [file 12889_2024_18222_MOESM1_ESM.docx]

# SUPPLEMENTARY MATERIALS

# Table S1. PRISMA checklist

| **Section and Topic** | **Item #** | **Checklist item** | **Location where item is reported** |
| --- | --- | --- | --- |
| **TITLE** | | |  |
| Title | 1 | Identify the report as a systematic review. | Page 1 |
| **ABSTRACT** | | |  |
| Abstract | 2 | See the PRISMA 2020 for Abstracts checklist. | Page 2 |
| **INTRODUCTION** | | |  |
| Rationale | 3 | Describe the rationale for the review in the context of existing knowledge. | Page 4-5 |
| Objectives | 4 | Provide an explicit statement of the objective(s) or question(s) the review addresses. | Page 5 |
| **METHODS** | | |  |
| Eligibility criteria | 5 | Specify the inclusion and exclusion criteria for the review and how studies were grouped for the syntheses. | Page 5-6 |
| Information sources | 6 | Specify all databases, registers, websites, organisations, reference lists and other sources searched or consulted to identify studies. Specify the date when each source was last searched or consulted. | Page 6 |
| Search strategy | 7 | Present the full search strategies for all databases, registers, and websites, including any filters and limits used. | Page 6 |
| Selection process | 8 | Specify the methods used to decide whether a study met the inclusion criteria of the review, including how many reviewers screened each record and each report retrieved, whether they worked independently, and if applicable, details of automation tools used in the process. | Page 6 |
| Data collection process | 9 | Specify the methods used to collect data from reports, including how many reviewers collected data from each report, whether they worked independently, any processes for obtaining or confirming data from study investigators, and if applicable, details of automation tools used in the process. | Page 6 |
| Data items | 10a | List and define all outcomes for which data were sought. Specify whether all results that were compatible with each outcome domain in each study were sought (e.g., for all measures, time points, analyses), and if not, the methods used to decide which results to collect. | Page 7 |
|  | 10b | List and define all other variables for which data were sought (e.g., participant and intervention characteristics, funding sources). Describe any assumptions made about any missing or unclear information. | Page 7 |
| Study risk of bias assessment | 11 | Specify the methods used to assess risk of bias in the included studies, including details of the tool(s) used, how many reviewers assessed each study and whether they worked independently, and if applicable, details of automation tools used in the process. | Page 6 |
| Effect measures | 12 | Specify for each outcome the effect measure(s) (e.g., risk ratio, mean difference) used in the synthesis or presentation of results. | N/A  (No meta-analysis was conducted) |
| Synthesis methods | 13a | Describe the processes used to decide which studies were eligible for each synthesis (e.g., tabulating the study intervention characteristics and comparing against the planned groups for each synthesis (item #5)). | N/A |
|  | 13b | Describe any methods required to prepare the data for presentation or synthesis, such as handling of missing summary statistics, or data conversions. | N/A |
|  | 13c | Describe any methods used to tabulate or visually display results of individual studies and syntheses. | Page 7 |
|  | 13d | Describe any methods used to synthesize results and provide a rationale for the choice(s). If meta-analysis was performed, describe the model(s), method(s) to identify the presence and extent of statistical heterogeneity, and software package(s) used. | N/A |
|  | 13e | Describe any methods used to explore possible causes of heterogeneity among study results (e.g. subgroup analysis, meta-regression). | N/A |
|  | 13f | Describe any sensitivity analyses conducted to assess robustness of the synthesized results. | N/A |
| Reporting bias assessment | 14 | Describe any methods used to assess risk of bias due to missing results in a synthesis (arising from reporting biases). | N/A |
| Certainty assessment | 15 | Describe any methods used to assess certainty (or confidence) in the body of evidence for an outcome. | N/A |
| **RESULTS** | | |  |
| Study selection | 16a | Describe the results of the search and selection process, from the number of records identified in the search to the number of studies included in the review, ideally using a flow diagram. | Figure 2 |
|  | 16b | Cite studies that might appear to meet the inclusion criteria, but which were excluded, and explain why they were excluded. | Table S7 |
| Study characteristics | 17 | Cite each included study and present its characteristics. | Table 1, page 7 |
| Risk of bias in studies | 18 | Present assessments of risk of bias for each included study. | Table S3 |
| Results of individual studies | 19 | For all outcomes, present, for each study: (a) summary statistics for each group (where appropriate) and (b) an effect estimate and its precision (e.g. confidence/credible interval), ideally using structured tables or plots. | N/A |
| Results of syntheses | 20a | For each synthesis, briefly summarise the characteristics and risk of bias among contributing studies. | Page 7-8 |
|  | 20b | Present results of all statistical syntheses conducted. If meta-analysis was done, present for each the summary estimate and its precision (e.g. confidence/credible interval) and measures of statistical heterogeneity. If comparing groups, describe the direction of the effect. | N/A |
|  | 20c | Present results of all investigations of possible causes of heterogeneity among study results. | N/A |
|  | 20d | Present results of all sensitivity analyses conducted to assess the robustness of the synthesized results. | N/A |
| Reporting biases | 21 | Present assessments of risk of bias due to missing results (arising from reporting biases) for each synthesis assessed. | N/A |
| Certainty of evidence | 22 | Present assessments of certainty (or confidence) in the body of evidence for each outcome assessed. | N/A |
| **DISCUSSION** | | |  |
| Discussion | 23a | Provide a general interpretation of the results in the context of other evidence. | Page 13-15 |
|  | 23b | Discuss any limitations of the evidence included in the review. | Page 15 |
|  | 23c | Discuss any limitations of the review processes used. | Page 15 |
|  | 23d | Discuss implications of the results for practice, policy, and future research. | Page 15 |
| **OTHER INFORMATION** | | |  |
| Registration and protocol | 24a | Provide registration information for the review, including register name and registration number, or state that the review was not registered. | Page 5 |
|  | 24b | Indicate where the review protocol can be accessed, or state that a protocol was not prepared. | Page 5 |
|  | 24c | Describe and explain any amendments to information provided at registration or in the protocol. | Page 5 |
| Support | 25 | Describe sources of financial or non-financial support for the review, and the role of the funders or sponsors in the review. | Page 16 |
| Competing interests | 26 | Declare any competing interests of review authors. | Page 17 |
| Availability of data, code and other materials | 27 | Report which of the following are publicly available and where they can be found: template data collection forms; data extracted from included studies; data used for all analyses; analytic code; any other materials used in the review. | Page 7 |

#

# Table S2. Search platforms and search terms

| **Type of literature** | **Search platforms** | **Date last searched** | **Search terms** |
| --- | --- | --- | --- |
| Published literature | Three electronic databases (Pubmed, EMBASE, Web of Science) | April 2022 | SHS [TIAB] OR "smoke pollut*" [TIAB] OR "smoking exposure" [TIAB] OR "secondhand smok*" [TIAB]OR "second-hand smok*" [TIAB]OR "passive smok*" [TIAB]OR "household smok*" [TIAB]OR "involuntary smok*" [TIAB]OR "environmental smok*" [TIAB]OR "environmental tobacco smoke" [TIAB]OR ETS [TIAB]OR "sidestream smok*" [TIAB]OR "mainstream smok*" [TIAB]OR tobacco smoke pollution [MeSH Terms] |
|  |  |  | advert* [TIAB] OR campaign* [TIAB] OR marketing [TIAB] OR promotion* [TIAB] OR "mass media" [TIAB] OR "social media" [TIAB] OR television [TIAB] OR radio [TIAB] OR billboard [TIAB] OR newspaper* [TIAB] OR multimedia [TIAB] OR website* [TIAB] OR online [TIAB] OR "internet" [TIAB] OR "Mass Media" [MeSH Terms] OR "Blogging" [MeSH Terms] OR "Social media" [MeSH Terms] OR "Pamphlets" [MeSH Terms] OR "Internet" [MeSH Terms] OR "Advertising" [MeSH Terms] OR "Marketing of Health Services" [MeSH Terms] OR "Social Marketing" [MeSH Terms] |
|  |  |  | polic* [TIAB] OR smoke-free [TIAB] OR smokefree [TIAB] OR antismoking [TIAB] OR law [TIAB] OR laws [TIAB] OR legislation* [TIAB] OR association [TIAB] OR evaluat* [TIAB] OR intervention [TIAB] OR efficacy [TIAB] OR effect [TIAB] OR attitude [TIAB] OR "health promotion" [TIAB] OR knowledge [TIAB] OR recommend* [TIAB] OR "health education" [TIAB] OR "public opinion*" [TIAB] OR "Smoking prevention" [MeSH Terms] OR Policy [MeSH Terms] OR "health promotion" [MeSH Terms] OR "Health education" [MeSH Terms] |
| Grey Literature | Google and google scholar | November 2022 | allintext: "second hand" AND smoking AND campaign |
|  |  |  | "passive" AND smoking AND campaign |
|  | YouTube |  | smokefree campaign OR second hand smoking campaign |
|  | Twitter |  | (second hand smoking campaign) OR (second hand smoke campaign) OR (passive smoking campaign) |
|  | Factiva |  | smoke-free campaign' OR 'secondhand smoking campaign' OR 'second-hand smoking campaign' |

# Table S3. Risk of bias assessment for each study

| **Cross-sectional studies** | **Turner (2021)** | **Zhang (2018)** | **Ravens (2016)** | **Alwan (2011)** | **Allmark (2012)** |
| --- | --- | --- | --- | --- | --- |
| **1. Was the study's target population representative of the national population in relation to relevant variables?** *2 = Representative or province/region or country; 1 = otherwise* | 1 | 2 | 1 | 1 | 1 |
| **2. Was the sample representative of the target population?** *2 = Probability sampling; 1 = otherwise* | 1 | 1 | 1 | 1 | 1 |
| **3. Was analysis conducted with sufficient coverage of the identified sample?** *2 = Response rate >= 75%; 1 = otherwise* | 1 | 1 | 1 | 1 | 2 |
| **4. Were the study subjects and the setting described in detail?** *1 = Reporting of descriptive statistics to describe the sample (i.e., age, sex etc.); 0 = otherwise* | 1 | 0 | 1 | 1 | 0 |
| **5. Was the condition of interest (exposure and outcome) measured in a valid and reliable way?** *2 = trained interviewer, administrative records; 1 = otherwise* | 2 | 2 | 2 | 1 | 2 |
| **6. Were confounding factors identified?** *1 = yes; 0 = otherwise* | 0 | 1 | 1 | 0 | 0 |
| **7. Was appropriate statistical analysis used and reporting?** *1 = yes; 0 = otherwise* | 1 | 1 | 1 | 0 | 0 |
| **TOTAL (out of 11)** | 7 | 8 | 8 | 5 | 6 |
|  |  |  |  |  |  |
| **Cohort studies** | **Niederdeppe (2007)** |  |  |  |  |
| **1. Were the two groups similar and recruited from the same population?** *1 = yes; 0 = otherwise* | 1 |  |  |  |  |
| **2. Were the exposures measured similarly to assign people to both exposed and unexposed groups?** *1 = yes; 0 = otherwise* | 1 |  |  |  |  |
| **3. Was the exposure and outcomes measured in a valid and reliable way?** *1 = yes; 0 = otherwise* | 0 |  |  |  |  |
| **4. Were confounding factors identified?** *1 = yes; 0 = otherwise* | 1 |  |  |  |  |
| **5. Were the groups/participants free of the outcome at the start of the study (or at the moment of exposure)?** *1 = yes; 0 = otherwise* | 1 |  |  |  |  |
| **6. Was the follow up time reported and sufficient to be long enough for outcomes to occur?** *1 = yes; 0 = otherwise* | 1 |  |  |  |  |
| **7. Was follow up complete, and if not, were the reasons to loss to follow up described and explored?** *1 = yes; 0 = otherwise* | 0 |  |  |  |  |
| **8. Were strategies to address incomplete follow up utilized?** *1 = yes; 0 = otherwise* | 0 |  |  |  |  |
| **9. Was appropriate statistical analysis used?** *1 = yes; 0 = otherwise* | 1 |  |  |  |  |
| **TOTAL (out of 9)** | 6 |  |  |  |  |
|  |  |  |  |  |  |
| **Qualitative studies** | **Rowa-Dewar (2016)** | **Rosemary (2021)** |  |  |  |
| **1. Clear statement on philosophical or theoretical premises in the study?** *1 = yes; 0 = otherwise* | 0 | 0 |  |  |  |
| **2. Is the study methodology appropriate for addressing research question?** *1 = yes; 0 = otherwise* | 1 | 1 |  |  |  |
| **3. Congruity between research methodology and the methods used to collect data?** *1 = yes; 0 = otherwise* | 1 | 1 |  |  |  |
| **4. Are the data analysed and represented in ways that are congruent with the stated methodological position?** *1 = yes; 0 = otherwise* | 1 | 0 |  |  |  |
| **5. Are the results interpreted in ways that are appropriate to the methodology?** *1 = yes; 0 = otherwise* | 1 | 1 |  |  |  |
| **6. Are the researcher's beliefs and values, and their potential influence on the study declared?** *1 = yes; 0 = otherwise* | 0 | 0 |  |  |  |
| **7. Influence of the researcher on the research, and vice-versa, is addressed?** *1 = yes; 0 = otherwise* | 0 | 0 |  |  |  |
| **8. Representation of participants and their voices?** *1 = yes; 0 = otherwise* | 1 | 0 |  |  |  |
| **9. Ethical approval by an appropriate body?** *1 = yes; 0 = otherwise* | 1 | 0 |  |  |  |
| **10. Relationship of conclusions to analysis, or interpretation of the data?** *1 = yes; 0 = otherwise* | 1 | 1 |  |  |  |
| **TOTAL (out of 10)** | 7 | 4 |  |  |  |

# Table S4. Sample characteristics of included studies from peer-reviewed literature (n=14) characteristics

| **Study** | **Campaign** | **Country** | **Campaign aim** | **Year** | **Scale^a^** | **Type of evaluation reported** | **Media details** | **Dissemination or activities** | **Results** | **Responsible agency/ organization** | **Risk of bias** |  |
| --- | --- | --- | --- | --- | --- | --- | --- | --- | --- | --- | --- | --- |
| Carroll (2018) | Women create smoke-free homes | Vietnam | To protect women and children from exposure to SHS throughout Vietnam | 2016-17 | C | Delivery,  Engagement,Awareness, Knowledge,Attitudes, and Intentions, Initiate change, Behavioural change | 30 sec adverts featuring a personal story of 41-y/o lung cancer victim and a call for action to "*Protect yourselves and your loved ones*" | Three 30-sec ads launched on Facebook, radio, print, online, and television. | Ads received > 4 million views. Evaluation study found 8% recalled the ads unprompted. 74% of females who does not smoke (and 75% males who smoke) tried to make their home 'smokefree'. 77% tried to persuade others to quit smoking after seeing the ads. 67% males who smoke reported a quit attempt after seeing ads. | The Vietnam Women's Union | Not peer reviewed (high) |  |
| Jin (2018) | National Tobacco Control Mass Media Campaign | China | To improve knowledge about the health effects of smoking and SHS exposure and shape attitudes and behaviours toward tobacco control policies | 2014 | C | Delivery, Knowledge,Attitudes, and Intentions, Initiate change | Adverts depicts how SHS affects other people in an office environment. | National Television Channels, 6 provincial satellite networks, and social media platforms | Campaign reached 24% of Chinese population. Evaluation study showed people gained knowledge on the harmful effects of smoking (not exposed: 73%, exposed 85%, p< 0.05). Individuals who smoked were more likely to reduce smoking (exposed: 13%, not exposed: 7%, p<0.05). The support for bans on smoking in public places also increased after the campaign, particularly among those who doesn’t smoke and females (data not presented). | Chinese Center for Health Education | Not peer reviewed (high) |  |
| Turner (2021) | Take it right outside (TiRO) | Scotland | To reduce the exposure of SHS among children by targeting parents who smoke at home and promote a change in their smoking behavior to create smoke-free home | 2014 | C | Behavioural change | Adverts depicting the dangers of SHS (i.e., SHS can continue for up to 5 hr after a cigarette has been extinguished in the home) | Television, radio, online,  print platforms, and a series of events in shopping centres | SHS exposure among children decreased by half from 12% in 2012 to 6% in 2015; There was a decrease relative to the underlying slope of hospital admissions for asthma (-0.48%, (-0.85 to -0.12)), p = 0.0096) in younger children but not older children | Scottish Government | Score: 7 out of 11 (medium) |  |
| Rowa-Dewar (2016) | Take it right outside (TiRO) | Scotland | To increase the awareness of SHS and the importance of smokefree homes to protect child health | 2014 | C | Delivery, Awareness, Knowledge,Attitudes, and Intentions, Initiate change | Adverts depicting the dangers of SHS (i.e., SHS can continue for up to 5 hr after a cigarette has been extinguished in the home) | Television, radio, online, community events, posters and a website | The socially disadvantaged parents had a high recall (n=13/17) and comprehension towards the campaign. Most parents said they would like to quit or have switched to other methods of nicotine consumption e.g. e-cigarettes, some reported going outside to smoke but it was not always feasible, no quantitative results reported. | Scottish Government | Score: 6 out of 9 (medium) |  |
| Zhang (2018) | Tips From Former Smokers | USA | To encourage smoking cessation | 2013-14 | C | Delivery, Initiate change | The 2013 and 2014 Phase 1 Spanish language adverts focussed on the health effects of SHS. | Television | Significant association between TV Gross Rating Points and increased call volume to Spanish-language Quitline (B=0.56 (0.45, 0.67), p<0.001) | The Centers for Disease Control and Prevention | Score: 8 out of 11 (low) |  |
| Prabandari (2018) | PEER Health project | Indonesia (North Lombok, Bima) | To reduce SHS exposure among pregnant women through smoke-free home campaign | 2015-18 | A | Delivery, Knowledge,Attitudes, and Intentions, | No details were specified for the media campaign. Community commitment to make all pregnant mothers' house smoke-free | Media campaigns, household education, smoke-free home commitment | Majority (no data presented) of pregnant women husband committed towards not smoking at home. | District health offices | Not peer reviewed (high) |  |
| Rutter (2018) | Secondhand smoke is poison | England | To raise awareness about the importance of smoking outside the home and protecting children from SHS | 2017 | A | Delivery | “*Secondhand smoke is poison*” campaign with a hard-hitting message around taking smoking right outside the home and the importance of protecting children from exposure | Not specified | Overall smoking has declined by 41% in this decade with smoke-free laws in place. No results reported on pre-post after the campaign. | Fresh - the North East's regional comprehensive tobacco control programme | Not peer reviewed (high) |  |
| Sahačić (2018) | Smeta Mi | Bosnia and Herzegovina | To raise awareness about tobacco harms (SHS harms) to ensure protection of human rights | 2016-17 | C | Delivery, Engagement | Social media and creative ways including letters from children to their parents demanding their right to clean air and photo campaign for support of new law | Social media | Campaign reached more than 3 million people especially to vulnerable populations, received more than 400 media coverage, 16K followers on Facebook. | Association PROI | Not peer reviewed (high) |  |
| Rayens (2016) | Rural Smoke-free Communities Project | USA | This project is part of the broader Rural Smoke-free Communities Project to assess the impact of a stage-specific, tailored community-based policy development intervention to promote smoke-free public policies | 2010-11 | A | Delivery, Awareness | Adverts featuring the use of local data, personal stories, and pictures of local citizen | Print adverts, direct mail postcards, newspapers, billboards, posters displayed in local businesses | People are more likely to recall gain framed ads (e.g., adverts that portrayed the benefits of smoke-free air) (29%) than loss-framed ads (e.g., adverts that discussed on the dangers of SHS) (20%). | Rural Smoke-free Communities Project | Score: 8 out of 11 (low) |  |
| Alwan (2011) | Smoke-Free Homes (SFH) | England (South Leeds) | To increase awareness of the health hazards of SHS and self-efficacy in being able to restrict smoking in homes using children as the primary change agent | 2003 | A | Delivery, Knowledge,Attitudes, and Intentions, Behavioural change | SFH toolkits, educational material | Educational activities using a SFH toolkit, promise forms containing messages on hazards of SHS, organized community-based events | Increase in smoke-free homes (pre: 35%, post: 68%), or smoking only allowed in a specific room (pre:12%, post: 23%), a reduction in smoking in the presence of children (pre:25%, post: 10%), keeping smoke-free promise (90% of the households) and reduction in smoking (pre: 54%, post: 48%). | West Yorkshire Smoking & Health | Score: 5 out of 11 (medium) |  |
| Allmark (2012) | Smoke-Free Home initiative | England (Rotherham) | To protect those exposed to second-hand smoke in private spaces. | 2009 | A | Delivery, Knowledge,Attitudes, and Intentions, Behavioural change | Information booklet and a bag containing items such as stickers, air fresheners, crayons and leaflets | Households signing up to smoke-free home initiative | Increase in smoke-free homes (pre: 60.8%, post: 92.9%), reduction in where smoking is allowed in most rooms (pre:6.1%, post: 0%). | NHS Rotherham | Score: 6 out of 11 (medium) |  |
| Niederdeppe (2008) | The Wisconsin Tobacco Prevention and Control Program | USA (Wisconsin) | To increase the volume of calls to the Wisconsin Tobacco Quit Line, raise awareness of the effects of SHS, and promote quit attempts | 2002-03 | S | Delivery, Awareness, Knowledge,Attitudes, and Intentions, Initiate change, Behavioural change | SHS adverts featured short vignettes from individuals affected by SHS | Television | SHS adverts recall were associated with higher awareness of the dangers of the harmful effects of breathing in SHS (OR = 2.1, 95% CI = 1.1-4.0) and higher awareness that SHS is as dangerous as people make it out to be (OR = 1.6, 95% CI = 1.0-2.7). SHS advert recall was not associated with quit attempts and smoking abstinence at 1 year. | The Wisconsin Tobacco Prevention and Control Program | Score: 6 out of 9 (medium) |  |
| Rosemary (2021) | #Suaratanparokok | Indonesia | #Suaratanparokok is a movement to inspire other people by speaking up about the need to quit smoking. | 2014 | C | Delivery,  Engagement,Awareness | Public Service Advertisements featuring the deleterious effects of SHS through personal testimonials | Social media | Viewers prefer and respond positively to the PSAs, even though engagement is low compared to other commercial adverts. Specifically, video that described the impact of SHS ("*Bahaya Paparan Asap Rokok - Ibu Ike*") received the highest number of views (>300k views), video raising awareness through personal testimonials ("In memoriam: Robby") has the highest number of engagements. | Ministry of Health and Vital Strategies Indonesia | Score: 4 out of 10 (medium) |  |
| Andreeva (2020) | Secondhand smoke awareness campaign | Ukraine | To raise awareness regarding the dangers of SHS exposure and to encourage activities able to diminish smoking in smoke-free zones | 2019 | C | Delivery, Knowledge,Attitudes, and Intentions, Initiate change, Behavioural change | Not specified | Not specified | Self-reported active protests against smoking in smoke-free places including making a comment to the person who smokes, posting a sign and reporting to the authorities (pre: 24.5%, post: 30.2%). Those who smoked felt increased pressure from others when they smoked in their homes (pre: 27.8%, post: 37.1%), work (pre: 14.9%, post: 23.6%), public places like cafes, bars, restaurants (pre: 13.9%, post: 25.1%), public transport stops (pre: 11.5%, post: 23.6%). The proportion of those who are currently smoking decreased significantly in the regions that were covered by media campaign (data not presented). | Not specified | Not peer reviewed (high) |  |
| *^a^G: Global; C: Country-level; S: State-level; A: Area/District/County-level* | | | | | | | | | | | | |

# Table S5. Mass media campaigns from the grey literature (n=61)

|  | | | | | | | | | |
| --- | --- | --- | --- | --- | --- | --- | --- | --- | --- |
| **Campaign** | **Country** | **Campaign aim** | **Year** | **Scale** | **Type of evaluation reported** | **Media details** | **Dissemination or activities** | **Metrics** | **Responsible agency/ organization** |
| **2022 campaigns (n=9)** | | | | | | | | | |
| Together Towards a Tobacco-Free Abu Dhabi | United Arab Emirates (Abu Dhabi) | To raise awareness about the harmful effects of smoking, the importance of quitting smoking tobacco, avoiding exposure to passive smoking, and to adopt a healthy lifestyle | 2022 | A | Delivery, Engagement | Post about how smoking affects people. "*Don’t smoke and you won’t regret it. Together towards a tobacco-free Abu Dhabi*" | Social media | The post on Twitter received around 82 likes, 31 retweets. | Abu Dhabi Public Health Centre |
| World No Tobacco Day (Cuba) | Cuba | To draw attention on the exposure to SHS, as well as to dissuade consumption in any of its forms | 2022 | C | Delivery | Not specified | Not specified | Not specified | Ministry of Health |
| Keep your family safe and keep smoke away from them | Jordan | To raise awareness of the dangers of smoking and SHS on physical health | 2022 | C | Delivery | Health messages calling on parents to adopt the idea of ​​quitting smoking to preserve their health and the health of their families and to appear as a good example for their children, number of media interviews with specialists to highlight the harms of smoking and passive smoking. | Social media | Not specified | Ministry of Health and International Relief Committee |
| Smoke-Free Bengaluru Initiative | India (Bengaluru) | This initiative focused on creating awareness about harmful effects of SHS and reduction in public place smoking | 2022 | A | Delivery | Not specified | Radio, school and college visits to raise awareness | Not specified | Bruhat Bengaluru Mahanagara Palike and Vital Strategies |
| Summer passive smoking prevention campaign | Japan (Chiba) | To encourage those who smokes to be considerate of the people around them and to foster momentum for passive smoking prevention throughout the prefecture. | 2022 | A | Delivery | Wetwipes containing messages about "Don't let the people around you breathe in the smoke" and that "Smoking is prohibited indoors in many facilities" | Distribution of wetwipes in medical facilities and public health centers | Not specified | Department of Health and Welfare |
| Time to quit campaign | Qatar | To raise the awareness in the community about the importance of quitting smoking, and the effects of active and passive smoking | 2022 | C | Delivery | Not specified | Direct and Virtual platforms – Campaign incorporated events, workshops, awareness competitions | Not specified | Qatar Cancer Society |
| World No Tobacco Day (Luxembourg) | Luxembourg | To raise public awareness of the many harmful effects of SHS | 2022 | C | Delivery | Not specified | Social media | Not specified | Ministry of Health |
| Giving Cigarettes Is Giving Harm | China | To increase knowledge of the harms of smoking and exposure to SHS and to actively discourage the practice of giving cigarettes as gifts to family and friends for Chinese New Year. | 2022 | C | Delivery, Engagement | This 45-sec advert actively discourage the practice of giving cigarettes as gifts to family and friends for Chinese New Year by discussing the harms of smoking and SHS | Social media, television | The adverts were viewed 352 times | China CDC and Vital Strategies |
| Smoke-free campaign | Nepal (Lalitpur) | To make people aware of the risks of smoking and the adverse effect smoking in public spaces | 2022 | A | Delivery | 60 flex board with different messages to discourage smoking in public | Billboards | Not specified | Lalitpur Metropolitan City Council |
| **2021 campaigns (n=14)** | | | | | | | | | |
| Drive Smokefree for Tamariki | New Zealand | To deliver an empowering message to promote smokefree cars. It aims to change the culture of smoking in cars with kids by reinforcing social norms, with the goal to reduce prevalence. | 2021 | C | Delivery, Engagement | Adverts that encourage those who smoke to go smokefree in their cars, and to do it for their kids and family, and to inform the public about the new smoke-free policy in vehicles | Online, radio, print, social media | Both adverts were collectively viewed more than 39k times | New Zealand Health Promotion Agency |
| World No-Smoking Day Cancer Prevention | Belarus | To raise public awareness about the harmful health effects of tobacco consumption and exposure to SHS | 2021 | C | Delivery | Informational and educational materials on tobacco products, quit smoking, passive smoking | Information, educational materials, leaflets, booklets, videos | Not specified | Republican Center for Hygiene, Epidemiology and Public Health |
| Secondhand Smoke. Never See You Again (二手煙．不再見) | Hong Kong | To educate citizens about the dangers of smoking and encourage those who smoke to quit smoking as soon as possible | 2021 | C | Delivery, Engagement, Knowledge, Attitudes, and Intentions | The Facebook page is to encourage people to say no to second-hand smoke, and support those who smoke to quit smoking | Publicity events around major train station, social media | More than 500 citizens pledged to refuse second-hand smoke and called on relatives and friends around them to quit smoking. The Facebook page has 7.5k followers. | Hong Kong Council on Smoking and Health |
| Smokefree Sheffield: Secondhand Smoke campaign | England (Sheffield) | To highlight the health risks of passive smoking and to encourage people to make their home a smokefree space to protect their loved ones from harmful SHS. | 2021 | A | Delivery, Engagement | Animation to raise awareness of the dangers of second-hand smoke and demonstrate how there is no safe way to smoke at home. | Social media | The advert on YouTube received around 200 views | Smokefree Sheffield |
| MyHOUSE Program (Malaysia Home withOut Smoking Environment) | Malaysia | To promote a smoke-free environment in private dwelling, to protect family members and their visitors from SHS | 2021 | C | Delivery,  Initiate change | Participation of smoke-free homes into the MyHOUSE program | Website, social media | 107 homes have participated in MyHOUSE program | Ministry of Health, Malaysian Women’s action for tobacco control and health, National Cancer Society Malaysia |
| Turn your home into a 100% smoke-free environment (Convertí Tu Hogar en un ambiente 100% libre de Humo) | Argentina | To increase awareness about the dangers of inhaling tobacco smoke | 2021 | C | Delivery | Not specified | Website, poster | Not specified | Ombudsman’s Observatory of Addictions and Problematic Consumption |
| If You Care: Smoke-Free Car | Turkey | To protect children from the tobacco epidemic and the harms of tobacco | 2021 | C | Delivery, Engagement | This 40-sec advert show the danger smoking in indoor spaces, such as cars, poses to children’s health | Social media | The adverts were viewed 221 times | Vital Strategies |
| Shadow & Harms of SHS | Bangladesh | To show the dangers of SHS and inform the public of existing laws prohibiting smoking in public places. | 2021 | C | Delivery, Engagement | Two short clips – i) to show the dangers of SHS and inform the public of existing laws prohibiting smoking in public places ii) to show the health risks from SHS in the home. | Social media | Both adverts were collectively viewed around 5k times | Vital Strategies |
| Quit Smoking to Protect Your Loved Ones | Vietnam | To raise awareness about the harmful effects of tobacco on those who smoke and those around them and call on people to strictly comply with the law on places where smoking is prohibited | 2021 | C | Delivery | Campaign shares the powerful story of Mrs Le Thi Tinh, who developed lung cancer after regular exposure to SHS in her home | Television, social media (Smoke-free Vietnam Facebook page) | Not specified | The Vietnam Women’s Union and the Vietnam Tobacco Control Fund and Vital Strategies |
| Not Around Us campaign | Ireland (Wexford County) | To protect children and young people from SHS exposure and contribute to the de-normalisation of smoking for children and young people. | 2021 | A | Delivery | Not specified | Leaflet, guidance documents, pledge, signage, social media, website | Not specified | Wexford County Council, Ireland |
| Not Around Us campaign | Ireland (Meath County) | To protect children and young people from SHS exposure and contribute to the de-normalisation of smoking for children and young people. | 2021 | A | Delivery | Not specified | Leaflet, guidance documents, pledge, signage, social media, website | Not specified | Meath County Council, Ireland |
| Not Around Us campaign | Ireland (Clare County) | To protect children and young people from SHS exposure and contribute to the de-normalisation of smoking for children and young people. | 2021 | A | Delivery | Not specified | Leaflet, guidance documents, pledge, signage, social media, website, webinar | Not specified | Clare County Council, Ireland |
| Not Around Us campaign | Ireland (Louth County) | To protect children and young people from SHS exposure and contribute to the de-normalisation of smoking for children and young people. | 2021 | A | Delivery | Not specified | Leaflet, guidance documents, pledge, signage, social media, website | Not specified | Louth County Council, Ireland |
| Commit to Quit | Ethiopia | To raise awareness on the harmful and deadly effects of tobacco use, second hand smoke exposure and to discourage the use of tobacco in any form | 2021 | C | Delivery | Not specified | Not specified | Not specified | United Nations Ethiopia |
| **2020 campaigns (n=5)** | | | | | | | | | |
| Not Around Us campaign | Ireland (Limerick County) | To protect children and young people from SHS exposure and contribute to the de-normalisation of smoking for children and young people. | 2020 | A | Delivery | Not specified | Leaflet, guidance documents, pledge, signage, social media, website | Not specified | Limerick County Council, Ireland |
| Not Around Us campaign | Ireland (Galway County) | To protect children and young people from SHS exposure and contribute to the de-normalisation of smoking for children and young people. | 2020 | A | Delivery | Not specified | Leaflet, guidance documents, pledge, signage, social media, website | Not specified | Galway County Council, Ireland |
| MyFamily MySmoke | Malaysia | To promote the implementation of smoke-free homes, ultimately to develop and promote a national health campaign showing the real life journey of those who smoke in making their homes smoke-free by incorporating and supported by scientific data | 2020 | C | Delivery, Engagement | The campaign highlights the stories of 3 individuals who have made their homes smoke-free, talking about the process and the benefits | Website, Social media | The advert on YouTube received around 147 views | Newton-Ungku Omar Fund & Malaysian Industry-Government Group for High Technology (MiGHT) |
| 2020 Passive smoking prevention campaign 受動喫煙防止キャンペーン | Japan (Kanagawa) | To protect children from cigarette smoke | 2020 | A | Delivery, Engagement | Posters and videos about the harms of SHS | Poster will be posted on digital signage or paper posters at health and welfare offices and centers in the prefecture, facilities in each municipality, and cooperating companies and organizations. | The advert was viewed 1558 times | Health and Welfare Offices, Municipalities offices, private organizations, hospital, preventative medical associations, and health foundations |
| Smokefree Homes | England (Walsall) | To protect children from the harms of SHS and to improve the health of families by not smoking | 2020 | A | Delivery, Engagement | Two short clips- i) an interview between a person who smoke and his GP, patient raising the concern about SHS while the GP listing the benefits associated with quitting smoking ii) warns of fire risk to life of smoking in the home, and the dangers of SHS | Social media | Both adverts were collectively viewed around 1k times | Walsall Public Health, Walsall CCG, Wasall Primary Care Network representatives |
| **2019 campaigns (n=13)** | | | | | | | | | |
| Aboriginal smoke-free home campaign | Australia (New South Wales) | To reduce smoking around the home and to prevent exposure to SHS. | 2019-20 | A | Delivery, Engagement | A 45-second cartoon highlighted three main messages: i) Don’t smoke close to the home ii) Smoke drifts further than you think iii) Your smoke harms others. | Social media, websites, cinemas, GP waiting rooms, local buses, train and bus stops and in Aboriginal community health centres | The advert on YouTube received more than 1.8k views | Western Sydney Local Health District |
| Second-hand smoke kills | Myanmar (Yangon) | To raise awareness of SHS and to remind the public that smoking in public establishment is not allowed | 2019 | A | Delivery | Messages and posts about the dangers of SHS | Social media | Not specified | Non-governmental People’s Health Foundation and Ministry of Health and Sports |
| Speak out: Express your right | Malaysia | To allow those who do not smoke to express their rights, and to convince those who smoke to quit smoking | 2019-24 | C | Delivery | Not specified | Website, poster | Not specified | Ministry of Health |
| Karlovy Vary Smoke free city campaign | Czechia (Karlovy Vary) | Part of this program will deploy a campaign to raise awareness on harmfulness of smoking and passive smoking for residents and visitors | 2019 | A | Delivery | Not specified | Not specified | Not specified | Karlovy Vary City Council |
| “Smokefree Home project. Stop destroying your child’s dream” | Thailand | To promote social awareness of the dangers of SHS in homes and to encourage those who smoke to quit for the benefits of the family, especially young children. | 2019 | C | Delivery | To prevent young children from exposure to second-hand smoke at home | Television, radio, print, outdoor billboards, online advertising, and transit advertising | Not specified | Action on Smoking and Health (ASH Thailand) |
| Smoke is an invisible killer (Дым – невидимый убийца) | Ukraine (Kyiv) | To warn the public about the dangers of passive smoking | 2019 | A | Delivery | Not specified | Videos, posters, and visuals on social media | Not specified | Kyiv City Council |
| World No Tobacco Day 2019 | Global | To raise awareness on the risks posed by tobacco smoking and SHS exposure, particular dangers of tobacco smoking to lung health, and the implications of second-hand exposure for lung health of people across age groups; | 2019 | G | Delivery | Not specified | Not specified | Not specified | World Health Organisation (WHO) |
| #PipeDown | Nigeria (Abuja) | To educate people, especially those who smoke, of the implication of smoking the flavored tobacco product on their own health and those who are around them | 2019 | A | Delivery | Posters carrying messages such as ‘smoking shisha for one hour is as bad as smoking 100 cigarettes’ and ‘smoking shisha in public places endangers others. The Instagram page provides users with the relevant information and facts on the dangers of Shisha smoke. | Lowering the prices of the burgers for one-week, social media | Not specified | Abuja leading Burger restaurant, Oliver’s Burger, and a public strategy firm, Gatefield |
| Ngamari Free – Second Hand Smoke and Your Health | Australia (Western Australia) | To reduce the impact of smoking on the health and wellbeing of all Aboriginal people in the Wheatbelt | 2019 | A | Delivery, Engagement | a 2m 20sec video clip to educate community on the effects of passive smoking and help encourage smoke free environments | Social media | The advert on YouTube received around 2k views | Western Australia Country Health Service |
| Say No to Bidis Campaign | Bahamas | To raise awareness to the harmful and deadly effects of tobacco use and SHS exposure, and to discourage the use of tobacco in any form | 2019 | C | Delivery | Not specified | Not specified | Not specified | Bahamas Government |
| Commit to Quit tobacco cessation campaign | Trinidad and Tobago | To encourage those who smoke to quit tobacco and to address SHS and to introduce the public the Tobacco Control Act and the penalties for offenders | 2019 | C | Delivery | Not specified | Billboards, shopping cart advertisements, newspaper inserts, and posters being disseminated throughout Trinidad and Tobago. | Not specified | WHO PAHO and Ministry of Health of Trinidad and Tobago |
| Quit for your pets | New Zealand | To encourage New Zealanders to consider the effects of smoking on their pets with “Quit for your pets”, an integrated advertising campaign. | 2019 | C | Delivery, Engagement | A 2 minute film features the relationship between a man and his dog, with the dog’s health declining due to cancer linked with second-hand and third-hand smoke. | Social media | The advert on YouTube received around 530k views | Quitline NZ |
| Passive smoking campaign | Slovenia | To raise public awareness of the ban on smoking in vehicles in the presence of minors persons and the harmful effects of passive smoking | 2019 | C | Delivery | Not specified | Social media | Not specified | Ministry of Health, National Institute of Public Health, Police, non-governmental organisations, and the media |
| **2018 campaigns (n=7)** | | | | | | | | | |
| National tobacco control mass media campaign | India | To warn people about the deadly harms of exposure to SHS and to promote the Quitline number | 2018 | C | Delivery, Engagement | “Clinical”, a 30-second PSA, reveals how exposure to SHS causes stroke and heart disease among those who do not smoke and encourages those who smoke to protect others by quitting smoking | Television, radio, social media | The advert on YouTube received around 3k views | Ministry of Health and Family Welfare and Vital Strategies |
| #healthismyright #健康是我的权利 | China | To highlight the devastating health effects of indoor air pollution caused by cigarette smoking and legislation is needed to empower and protect the rights of those who do not smoke | 2018 | C | Delivery, Engagement | Short clip to show that indoor smoking can cause the PM2.5 indoor levels to skyrocket. | Social media | The advert on Weibo received more than 5.8 million views | World Health Organisation (WHO) China |
| A Life in Smoke | Bosnia and Herzegovina | To raise awareness about the harmful effects of tobacco smoke and the necessity to ban indoor smoking in all public places to protect children and those who choose not to smoke and creating an environment where they will not be the victims of tobacco smoke | 2018 | C | Delivery, Engagement | Not relevant | A two-day creative exhibit aimed at showing what the fight of citizens for clean air in public spaces full of tobacco smoke looks like | The two-day exhibit attracted over 10.000 people in attendance. | Klima bez dima initiative |
| Your home, my workplace (Jouw huis, mijn werkplek) | Belgium | To make passive smoking a subject of discussion and to protect their employees as well as possible against the consequences of passive smoking | 2018 | A | Delivery | Training video to teach employees how to talk to their customers who smoke, roll of peppermint to break the ice, manual for employers to help them develop a policy | Manual, training video and a roll of peppermints | Not specified | the Flemish Institute for Healthy Living and the Flemish government |
| Car without smoke. Protect them (En esto temenos nidos que estar todos Unidos nidos) | Spain | To end tobacco use in cars through schools visits throughout Spain to turn minors into “health agents” and ensure that their parents do not smoke inside their vehicles | 2018 | C | Delivery | Badges that identifies “smoke-free cars” | Not specified | Not specified | The General Nursing Council |
| Secondhand smoke can kill your pet (Passivrauchen kann Ihr Haustier töten) | Austria | To raise awareness about the harms associated with passive smoking to animals | 2018 | C | Delivery | Not specified | Not specified | Not specified | Austrian Animal Welfare Association |
| #Respect | Vietnam | To respect the law on tobacco control and respect own and others’ health by ending indoor smoking at public spaces to prevent exposure to second-hand smoke | 2018 | C | Delivery | The hashtag #Respect was created to help people spread anti-smoking messages on social networks and the public is encouraged to support the campaign by sharing posts, making supportive comments and creating tobacco-related stories with the hashtag. | Social media | Not specified | Campaign for Tobacco-Free Kids (TFK), Community Research and Development Services (CDS), the Development and Policies Research Center (DEPOCEN), Hanoi University of Public Health (HUPH), HealthBridge Vietnam, Thuongmai University, Vietnam Public Health Association (VPHA) and Vital Strategies. |
| **2017 campaigns (n=7)** | | | | | | | | | |
| Breathe Free (Շնչենք ազատ) | Armenia | To raise public awareness of the many harmful effects of SHS on the health | 2017 | C | Delivery | Leaflets about smoking and the harmful effects of smoke, also called on them to replace their cigarettes with surprise gifts | Leaflets | Not specified | Armenian Relief Fund |
| Stop Tobacco Bangladesh | Bangladesh | To encourage those who smoke to quit and to protect those who do not smoke (e.g., children) by showing the real health harms of exposure to SHS | 2017-Ongoing | C | Delivery, Engagement | 30-sec PSA advert (“Sickening”) telling the story of a young boy who developed asthma. The PSA goes on to warn that exposure to SHS among those who do not smoke will increase the risk of lung cancer and tuberculosis. Social media content will also run on “Stop Bangladesh” Facebook page and Twitter account | Television, social media | The advert on YouTube received around 1k views. The Facebook page has 766k followers | Ministry of Health and Family Welfare and Vital Strategies |
| Smoke Free Home Campaign – EMBRACE | Cambodia (Choam Ksant) | To promote awareness about the harmful effects of tobacco and SHS affecting especially children, unborn babies, and pregnant women | 2017 | A | Delivery, Engagement | Not specified | Seminar and talks to the general public | Campaign reach up to 350 people | Global Affairs Canada, Provincial Health Department, Choam Ksant district |
| Smoke-Free Baguio’ campaign | Philippines | To encourage residents to shy away from cigarette smoking | 2017 | A | Delivery, Engagement | Facebook page sharing info about harms of second hand smoking to vulnerable population like children, and a place for those who do not smoke to express their right to breathe in clean air | Social media, a fun run, mural painting, education campaign | Campaign reach more than 6k followers on Facebook | Baguio City Health Office |
| Be Smoke-Free Tobacco Campaign | U.S. Virgin Islands | To discourage tobacco use among young people, promote quitting among those who smoke, eliminate exposure to SHS, increase awareness of the dangers of tobacco use, and to raise awareness to the Smoke-free law. | 2017 | C | Delivery | Public Service Advertisements | Television, social media, previews at the movie theatres, radio, PSAs, billboards placed throughout the territory | Not specified | Virgin Islands Department of Health |
| Secondhand Smoke is Poison | England (Northeast) | This campaign issued a plea to those who smoke but aren’t yet ready to quit to take it right outside and ensure loved ones such as children and grandchildren are not regularly exposed to smoke in indoor spaces. | 2017-18 | A | Delivery,  Awareness, Knowledge, Attitudes, and Intentions, Initiate change, Behavioural change | 40 sec ads and posters telling those who smoke to never smoke indoors for the sake of their kids | Television, radio, social media, leaflets and posters for partners, an intervention to help front line staff deliver messages around SHS to members of the public in a non-judgmental and effective way | 65% of people who smoke recalled the campaign, 92% found it believable, 66% made them more likely to quit, 75% were more concerned about smoking, and 38% took action, from cutting down, to going outside to smoke, stopping smoking in the same room as a family member, stopping smoking, or switching to an electronic cigarette | Fresh, UK's first dedicated regional tobacco control programme |
| “You smoke...they smoke” | Malta | To inform the public on the content of the new legislation and the dangers of SHS. | 2017 | C | Delivery | Not specified | Website, Pamphlet | Not specified | Government of Malta |
| **2016 campaigns (n=6)** | | | | | | | | | |
| Finish It campaign (#CATmageddon) | USA | To educate and raise awareness about the dangers of SHS on animals. | 2016 | C | Delivery, Engagement | A short video highlighting the doubled risk of cancer in cats exposed to second-hand smoke on YouTube | Social media | The advert on YouTube received more than 4 million views and it was tweeted more than 100k times, reaching more than 378 million people | Truth Initiative |
| Protect yourself and your loved ones | Vietnam (Hanoi) | To communicate the harmful effects of SHS | 2016 | A | Delivery | Short clips based on true story of Nguyễn Thị Hương, a non-smoking victim of lung cancer | Television, social media, newspapers | Not specified | Vietnam Women’s Union, Ministry of Health, Vital Strategies |
| Smoke-free on the road | Germany (Berlin) | To raise awareness that smoking in the car is particularly harmful to children | 2016 | A | Delivery | Not specified | Brochures, Stickers, Magazines, social media, support from pediatric practices | Not specified | Drug commissioners of the Federal Government, The Federal Center for Health Education (BZgA), the German Academy for Child and Adolescent Medicine (DAKJ), the German Medical Association, the National Association of Statutory Health Insurance Funds |
| Quit Smoking Now (Tuku Ifi Leva) | Tonga | To highlight the dangers of tobacco and aims to reduce the number of preventable deaths and disability inflicted on adults and children by tobacco. | 2016 | C | Delivery, Engagement | Advertisement showing the harms and dangers of second-hand smoking to children | Television, radio, press, social media, website | The advert on YouTube received around 1k views | Ministry of Health |
| Secondhand Smoke in Vehicles Prevention Campaign | USA (Gulf and Franklin County) | To prevent SHS in vehicles | 2016 | A | Delivery | Messages promoting the harm of SHS to children in vehicles | Posters and radio | Not specified | Florida Health |
| National Anti-Tobacco Campaign | Chad | To highlight the deadly harms of tobacco to especially children exposed to second-hand smoke (SHS) | 2016 | C | Delivery, Engagement | 30-sec PSA adverts; ("*Idrissa*") telling the story of a tailor who smoked and developed mouth cancer. The cancer was so bad that he was unable to eat outside because of his appearance. (“Cigarettes is eating your baby alive”) graphically shows that cigarette smoke contains poisons that can trigger severe health problems in those who do not smoke | Television and radio | The advert on YouTube received around 1.5k views | Government of the Republic of Chad |
| *^a^G: Global; C: Country-level; S: State-level; A: Area/District/County-level* | | | | | | | | | |

**Table S6. Social Media Campaign Engagement**

| **Campaign** | **Country** | **Platform** | **Views^a^** |
| --- | --- | --- | --- |
| MyFamilyMySmoke | Malaysia | YouTube | 147 |
| Smokefree Sheffield: Secondhand Smoke campaign | England | YouTube | 200 |
| If You Care: Smoke-Free Car | Turkey | YouTube | 221 |
| Giving Cigarettes Is Giving Harm | China | YouTube | 352 |
| Quit Smoking Now (Tuku Ifi Leva) | Tonga | YouTube | 1,000 |
| Smokefree Homes | England | YouTube | 1,000 |
| Stop Tobacco Bangladesh | Bangladesh | YouTube | 1,000 |
| National Anti-Tobacco Campaign | Chad | YouTube | 1,500 |
| 2020 Passive smoking prevention campaign 受動喫煙防止キャンペーン | Japan | Not reported | 1,558 |
| Aboriginal smoke-free home campaign | Australia | YouTube | 1,800 |
| Ngamari Free - Second Hand Smoke and Your Health | Australia | YouTube | 2,000 |
| National tobacco control mass media campaign | India | YouTube | 3,000 |
| Shadow & Harms of SHS | Bangladesh | Undisclosed social media | 5,000 |
| Drive Smokefree for Tamariki | New Zealand | Undisclosed social media | 39,000 |
| Suara tanpa rokok | Indonesia | YouTube | 35 to 526,921 |
| Quit for your pets | New Zealand | YouTube | 530,000 |
| ^a^Data was collected on 10 November 2022 | | | |

| **Table S7. Excluded studies and reasons of exclusion** | | | | |
| --- | --- | --- | --- | --- |
| **Author** | **Year** | **Title** | **Final decision** | **Reason** |
| Fairbrother | 2020 | Children's learning from a Smokefree Sports programme: Implications for health education | **EXCLUDE** | No intervention (mass media campaign or mass media component in the smoke-free policy) |
| Debchoudhury | 2019 | Implementing Smoke-Free Housing Policies Among Multiunit Housing Owners in New York City Between 2012 and 2015 | **EXCLUDE** | No intervention (mass media campaign or mass media component in the smoke-free policy) |
| Mantey | 2021 | Secondhand Smoke Exposure at Home and/or in a Vehicle: Differences Between Urban and Non-Urban Adolescents in the United States, From 2015 to 2018 | **EXCLUDE** | No intervention (mass media campaign or mass media component in the smoke-free policy) |
| McDaniel | 2018 | US Media Coverage of Tobacco Industry Corporate Social Responsibility Initiatives | **EXCLUDE** | No data on SHS |
| Heath | 2016 | Few U.S. schools of nursing on campuses with smoke-free policies: A Call for Action | **EXCLUDE** | No intervention (mass media campaign or mass media component in the smoke-free policy) |
| Yamato | 2020 | Secondhand Smoke from a Veranda Spreading to Neighboring Households | **EXCLUDE** | No intervention (mass media campaign or mass media component in the smoke-free policy) |
| Yunarman | 2020 | Compliance with Smoke-Free Policy and Challenges in Implementation: Evidence from Bengkulu, Indonesia | **EXCLUDE** | No intervention (mass media campaign or mass media component in the smoke-free policy) |
| Petersen | 2018 | An exploratory study of knowledge, attitudes, and beliefs related to tobacco use and secondhand smoke among women in Aleta Wondo, Ethiopia | **EXCLUDE** | No intervention (mass media campaign or mass media component in the smoke-free policy) |
| - | 2017 | Campaign smoke-free on the way" catches on! "Protect children from passive smoking": Marlene Mortler visits the Stadt Apotheke in Lauf a. d. Pegnitz" | **EXCLUDE** | Cannot locate PDF |
| Do | 2020 | Student Attitudes Toward Tobacco Use and Tobacco Policies on College Campuses | **EXCLUDE** | No intervention (mass media campaign or mass media component in the smoke-free policy) |
| Bartington | 2020 | Smoking behaviours and attitudes towards campus-wide tobacco control policies among staff and students: a cross-sectional survey at the University of Birmingham | **EXCLUDE** | No intervention (mass media campaign or mass media component in the smoke-free policy) |
| Cho | 2017 | Make the world beautiful and healthy by making your country smoke free: Case study between Iceland and Thailand? | **EXCLUDE** | No data on SHS |
| Stein | 2016 | The Experience With Smoke-Free Policies in Affordable Multiunit Housing in North Carolina: A Statewide Survey | **EXCLUDE** | No intervention (mass media campaign or mass media component in the smoke-free policy) |
| Uang | 2017 | Smokefree implementation in Colombia: Monitoring, outside funding, and business support | **EXCLUDE** | No intervention (mass media campaign or mass media component in the smoke-free policy) |
| Augustynowicz | 2018 | Health needs in local government policies in Poland in the context of anti-smoking health policy programs | **EXCLUDE** | No intervention (mass media campaign or mass media component in the smoke-free policy) |
| Wei | 2019 | Evaluation of the Effectiveness of Comprehensive Smoke-Free Legislation in Indoor Public Places in Shanghai, China | **EXCLUDE** | No intervention (mass media campaign or mass media component in the smoke-free policy) |
| Wahyuti | 2019 | Monitoring Compliance and Examining Challenges of a Smoke-free Policy in Jayapura, Indonesia | **EXCLUDE** | No intervention (mass media campaign or mass media component in the smoke-free policy) |
| Gentzke | 2018 | Attitudes and experiences with secondhand smoke and smoke-free policies among subsidised and market-rate multiunit housing residents living in six diverse communities in the USA | **EXCLUDE** | No intervention (mass media campaign or mass media component in the smoke-free policy) |
| Tran | 2021 | Secondhand smoke in public places in Vietnam: An assessment 5 years after implementation of the tobacco control law | **EXCLUDE** | No intervention (mass media campaign or mass media component in the smoke-free policy) |
| Guillaumier | 2017 | Australian university smoke-free policy implementation: a staff and student survey | **EXCLUDE** | No data on SHS |
| Cooper | 2016 | A baseline assessment of attitudes toward tobacco free campus policies in a U.S./México border university | **EXCLUDE** | No intervention (mass media campaign or mass media component in the smoke-free policy) |
| Duffy | 2019 | School gates - Tobacco and smoke free zones | **EXCLUDE** | No intervention (mass media campaign or mass media component in the smoke-free policy) |
| Hamada | 2021 | Status of smoke-free hospitals after implementation of the Hyogo Prefectural Ordinance on the Prevention of Expxosure to Secondhand Smoke and the Revised Health Promotion Act | **EXCLUDE** | No intervention (mass media campaign or mass media component in the smoke-free policy) |
| Kataoka | 2021 | Current status and intention to change indoor smoking rules by enforcing smoke-free legislation in Japan | **EXCLUDE** | No intervention (mass media campaign or mass media component in the smoke-free policy) |
| Ogah | 2016 | Awareness and perception of harmful effects of smoking in Nigeria: Findings from the Abia State, Nigeria NCD risk factors community based survey | **EXCLUDE** | No intervention (mass media campaign or mass media component in the smoke-free policy) |
| Azagba | 2019 | Exposure to secondhand smoke in vehicles among Canadian adolescents: Years after the adoption of smoke-free car laws | **EXCLUDE** | No intervention (mass media campaign or mass media component in the smoke-free policy) |
| Takenobu | 2022 | Impact of workplace smoke-free policy on secondhand smoke exposure from cigarettes and exposure to secondhand heated tobacco product aerosol during COVID-19 pandemic in Japan: the JACSIS 2020 study | **EXCLUDE** | No intervention (mass media campaign or mass media component in the smoke-free policy) |
| Bader | 2018 | The carrot not the stick: Creating incentives for voluntary enforcement of smoke-free policies through smoke-free zone certification | **EXCLUDE** | No intervention (mass media campaign or mass media component in the smoke-free policy) |
| Li | 2020 | Does smoke-free legislation work for teens too? A logistic regression analysis of smoking prevalence and gender among 16 years old in Ireland, using the 1995-2015 ESPAD school surveys | **EXCLUDE** | No intervention (mass media campaign or mass media component in the smoke-free policy) |
| Ikpe | 2018 | Exposure to second-hand smoke and support for smoke-free policies in an urban district in Lagos, Nigeria | **EXCLUDE** | No intervention (mass media campaign or mass media component in the smoke-free policy) |
| Jackson-Morris | 2019 | The contribution of a 'whole of government' smoke-free policy on the island of St Helena | **EXCLUDE** | No data on SHS |
| Ickes | 2017 | Students' Beliefs About and Perceived Effectiveness of a Tobacco-Free Campus Policy | **EXCLUDE** | No data on SHS |
| Berry | 2021 | A Longitudinal Assessment of Corrective Advertising Mandated in United States v. Philip Morris USA, Inc | **EXCLUDE** | Specific group recruited for intervention |
| F. Al-Jayyousi | 2021 | Students' perceptions of a university 'No Smoking' policy and barriers to implementation: a cross- sectional study | **EXCLUDE** | No intervention (mass media campaign or mass media component in the smoke-free policy) |
| Lee | 2017 | An Experiment Assessing Punitive versus Wellness Framing of a Tobacco-Free Campus Policy on Students' Perceived Level of University Support | **EXCLUDE** | No intervention (mass media campaign or mass media component in the smoke-free policy) |
| King | 2017 | Involving mosques in health promotion programmes: a qualitative exploration of the MCLASS intervention on smoking in the home | **EXCLUDE** | No intervention (mass media campaign or mass media component in the smoke-free policy) |
| Jin | 2020 | The Effects of Interactivity on the Attitudes and Intentions about Second-hand Smoke: Comparison of Two Mechanisms | **EXCLUDE** | No intervention (mass media campaign or mass media component in the smoke-free policy) |
| Bafunno | 2017 | What do the children think about smoking and how are they helped in prevention? | **EXCLUDE** | No intervention (mass media campaign or mass media component in the smoke-free policy) |
| Seitz | 2018 | Enforcement of Smoke/Tobacco-free Policies at Public Universities in the US | **EXCLUDE** | No data on SHS |
| Rungruanghiranya | 2017 | Impact of Tobacco Control Campaigns on Smoking Behaviors in Thai Medical Schools | **EXCLUDE** | No intervention (mass media campaign or mass media component in the smoke-free policy) |
| Prado-Galbarro | 2020 | Adolescent Tobacco Exposure in 31 Latin American Cities before and after the Framework Convention for Tobacco Control | **EXCLUDE** | No intervention (mass media campaign or mass media component in the smoke-free policy) |
| Park | 2019 | Attitudes of Korean smokers towards smoke-free public places: findings from the longitudinal ITC Korea Survey, 2005-2010 | **EXCLUDE** | No intervention (mass media campaign or mass media component in the smoke-free policy) |
| Nnadiwa | 2018 | Awareness and support for the statewide smoking law among cigarette smokers in Lagos State, Nigeria | **EXCLUDE** | No intervention (mass media campaign or mass media component in the smoke-free policy) |
| Martin | 2017 | Staff and patient perspectives of a smoke-free health services policy in South Australia: A state-wide implementation | **EXCLUDE** | No intervention (mass media campaign or mass media component in the smoke-free policy) |
| El-Awa | 2020 | The status of tobacco control in the Eastern Mediterranean Region: progress in the implementation of the MPOWER measures | **EXCLUDE** | No intervention (mass media campaign or mass media component in the smoke-free policy) |
| Bumb | 2017 | Workplace tobacco control promotion in India-a pilot study | **EXCLUDE** | No intervention (mass media campaign or mass media component in the smoke-free policy) |
| Dilliott | 2020 | The attitudes and behaviors of students, staff and faculty towards smoke-free and tobacco-free campus policies in North American universities: A narrative review | **EXCLUDE** | Review |
| Gupta | 2018 | Increasing smoke free public places & creating tobacco free educational institutions | **EXCLUDE** | No intervention (mass media campaign or mass media component in the smoke-free policy) |
| Razzak | 2021 | Creating smoke free community ensuring smoke free home | **EXCLUDE** | None of the campaign element were described. |
| Çobanoǧlu | 2020 | Assessment of the change in burden of respiratory diseases in children and household smoking habits in Turkey after adoption of National Tobacco Control Program | **EXCLUDE** | No intervention (mass media campaign or mass media component in the smoke-free policy) |
| Krishnan | 2018 | Malaysian health promotion board (MySihat) smoke-free city initiative | **EXCLUDE** | None of the campaign element were described. |
| Wilson | 2021 | A survey of the smokefree status of pedestrian-only spaces in 10 New Zealand local government areas | **EXCLUDE** | Campaigns that promote smoke-free law |
| Obeidat | 2016 | Public support for smoke-free policies in Jordan, a high tobacco burden country with weak implementation of policies: Status, opportunities, and challenges | **EXCLUDE** | No intervention (mass media campaign or mass media component in the smoke-free policy) |
| Zhou | 2019 | Effect of tobacco control policies on the Swedish smoking quitline using intervention time-series analysis | **EXCLUDE** | None of the campaign element were described. |
| Bhutia | 2021 | Health assembly of young leaders advocating for children's rights for tobacco free environment | **EXCLUDE** | No intervention (mass media campaign or mass media component in the smoke-free policy) |
| Thompson | 2019 | Implementing an Evidence-based Tobacco Control Program at Five 2-1-1 Call Centers: An Evaluation Using the Consolidated Framework for Implementation Research | **EXCLUDE** | Intervention study |
| Suteeerangkul | 2019 | The effects of Community Participation Program on smoke-free home in sub-urban community of Thailand | **EXCLUDE** | Intervention study |
| Gong | 2016 | Implementation of the Tobacco-Free Campus Policy on College Campuses: Evidence From a Survey of College Students in Beijing | **EXCLUDE** | Campus level campaign |
| Wray | 2021 | Effects of a campus-wide tobacco-free policy on tobacco attitudes, norms and behaviors among students, staff and faculty | **EXCLUDE** | Campus level campaign |
| Sendall | 2021 | University Staff and Students' Attitudes towards a Completely Smoke-Free Campus: Shifting Social Norms and Organisational Culture for Health Promotion | **EXCLUDE** | Campus level campaign |
| Correa-Fernández | 2017 | Implementation of a tobacco-free workplace program at a local mental health authority | **EXCLUDE** | Campus level campaign |
| Weinmann | 2018 | Developing an Awareness Campaign to Reduce Second Hand Smoke Among Disadvantaged Families-A Participatory M-Health Approach | **EXCLUDE** | Development of a campaign |
| Huong | 2021 | The lesson learnt from smoke-free restaurant model in Hoan Kiem, Vietnam | **EXCLUDE** | Campaigns that promote smoke-free law |
| Wen | 2018 | The role of social listening in promoting 100% smoke-free law in Shanghai | **EXCLUDE** | Campaigns that promote smoke-free law |
| Braverman | 2018 | Predicting students' noncompliance with a smoke-free university campus policy | **EXCLUDE** | Campus level campaign |
| Santo | 2017 | A Tobacco-Free Medical Campus Policy is Associated With Decreased Secondhand Smoke Exposure and Increased Satisfaction Among Military Medical Employees: Results of a Mixed-Methods Evaluation | **EXCLUDE** | Campus level campaign |
| Duan | 2020 | Secondhand smoke (SHS) exposure before and after the implementation of the Tobacco Free Cities (TFC) initiative in five Chinese cities: a pooled cross-sectional study | **EXCLUDE** | Campaigns that promote smoke-free law |
| Zhao | 2019 | Cigarette Smoking and Secondhand Smoke Exposure Before and After a Tobacco-Free Olympic Policy Period: Qingdao, China | **EXCLUDE** | Campaigns that promote smoke-free law |
| Chen | 2018 | International best practice adopted in China: Evidence-based mass media campaigns to support city-level 100% smoke free laws | **EXCLUDE** | Campaigns that promote smoke-free law |
| Pham | 2018 | Implementation of smoke free policy in restaurant setting - Experience of a NGO in Vietnam | **EXCLUDE** | Campaigns that promote smoke-free law |
| Khow | 2018 | Engaging new voices and key opinion leaders in support of smoke-free policies in China | **EXCLUDE** | Campaigns that promote smoke-free law |
| Skerletopoulos | 2020 | Trikala Quits Smoking": A Citizen Co-Creation Program Design to Enforce the Ban on Smoking in Enclosed Public Spaces in Greece" | **EXCLUDE** | Campaigns that promote smoke-free law |
| Goecke | 2018 | Tobacco prevention using the example of the smoke-free" campaign : Nationwide measures by the Federal Center for Health Education (BZgA) | **EXCLUDE** | Review (data extracted in grey lit) |
